# Lipid kinase PIP5Kα contributes to Hippo pathway activation via interaction with Merlin and by mediating plasma membrane targeting of LATS1

**DOI:** 10.1186/s12964-023-01161-w

**Published:** 2023-06-19

**Authors:** Truc Phan Hoang Le, Nga Thi Thanh Nguyen, Duong Duy Thai Le, Muhammad Ayaz Anwar, Sang Yoon Lee

**Affiliations:** 1 Department of Biomedical Sciences, Ajou University Graduate School of Medicine, Suwon, Gyeonggi 16499 Republic of Korea; 2 Department of Applied Chemistry, Kyung Hee University International Campus, Yongin, Gyeonggi 17104 Republic of Korea; 3 Institute of Medical Science, Ajou University School of Medicine, Suwon, Gyeonggi 16499 Republic of Korea

**Keywords:** PIP5Kα, Merlin, LATS1, PIP2, Hippo pathway, YAP/TAZ, Plasma membrane

## Abstract

**Background:**

The Hippo pathway plays a critical role in controlled cell proliferation. The tumor suppressor Merlin and large tumor suppressor kinase 1 (LATS1) mediate activation of Hippo pathway, consequently inhibiting the primary effectors, Yes-associated protein (YAP) and transcriptional coactivator with PDZ-binding motif (TAZ). Phosphatidylinositol 4,5-bisphosphate (PIP2), a lipid present in the plasma membrane (PM), binds to and activates Merlin. Phosphatidylinositol 4-phosphate 5-kinase α (PIP5Kα) is an enzyme responsible for PIP2 production. However, the functional role of PIP5Kα in regulation of Merlin and LATS1 under Hippo signaling conditions remains unclear.

**Methods:**

PIP5Kα, Merlin, or LATS1 knockout or knockdown cells and transfected cells with them were used. LATS1, YAP, and TAZ activities were measured using biochemical methods and PIP2 levels were evaluated using cell imaging. Low/high cell density and serum starvation/stimulation conditions were tested. Colocalization of PIP5Kα and PIP2 with Merlin and LATS1, and their protein interactions were examined using transfection, confocal imaging, immunoprecipitation, western blotting, and/or pull-down experiments. Colony formation and adipocyte differentiation assays were performed.

**Results:**

We found that PIP5Kα induced LATS1 activation and YAP/TAZ inhibition in a kinase activity-dependent manner. Consistent with these findings, PIP5Kα suppressed cell proliferation and enhanced adipocyte differentiation of mesenchymal stem cells. Moreover, PIP5Kα protein stability and PIP2 levels were elevated at high cell density compared with those at low cell density, and both PIP2 and YAP phosphorylation levels initially declined, then recovered upon serum stimulation. Under these conditions, YAP/TAZ activity was aberrantly regulated by PIP5Kα deficiency. Mechanistically, either Merlin deficiency or LATS1 deficiency abrogated PIP5Kα-mediated YAP/TAZ inactivation. Additionally, the catalytic domain of PIP5Kα directly interacted with the band 4.1/ezrin/radixin/moesin domain of Merlin, and this interaction reinforced interaction of Merlin with LATS1. In accordance with these findings, PIP5Kα and PIP2 colocalized with Merlin and LATS1 in the PM. In PIP5Kα-deficient cells, Merlin colocalization with PIP2 was reduced, and LATS1 solubility increased.

**Conclusions:**

Collectively, our results support that PIP5Kα serves as an activator of the Hippo pathway through interaction and colocalization with Merlin, which promotes PIP2-dependent Merlin activation and induces local recruitment of LATS1 to the PIP2-rich PM and its activation, thereby negatively regulating YAP/TAZ activity.

Video Abstract

**Supplementary Information:**

The online version contains supplementary material available at 10.1186/s12964-023-01161-w.

## Introduction

The evolutionarily conserved Hippo pathway serves as a primary signaling hub for regulating cell growth, proliferation, regeneration, and differentiation [1–3]. Activation of the Hippo pathway restricts cell proliferation by mediating cell contact-based inhibitory signaling (contact inhibition), thereby playing a pivotal role in maintaining normal tissue development [1–4]. Numerous studies have indicated the implications of Hippo pathway dysregulation in various pathological progressions, including tumorigenesis [3, 5]. The main components of the Hippo pathway are mammalian STE20-like protein kinase 1/2 (MST1/2), large tumor suppressor kinase 1/2 (LATS1/2), SAV1 and MOB1 (adaptors for MST1/2 and LATS1/2, respectively), Yes-associated protein (YAP) and its paralog transcriptional coactivator with PDZ-binding motif (TAZ), and transcription factor TEAD1–4 [2–4, 6]. Upon activation of the Hippo pathway, MST1/2 phosphorylates and activates LATS1/2, which then phosphorylates and inactivates the downstream effectors, YAP/TAZ [2–4]. Phosphorylated YAP/TAZ undergo cytoplasmic retention by binding to 14–3-3 proteins, or proteasomal degradation via polyubiquitination by the E3 ubiquitin ligase β-TrCP [3, 4, 7, 8]. When the Hippo pathway is turned off, MST1/2 and LATS1/2 are inactivated; thus, the transcriptional coactivators YAP/TAZ become activated, allowing their nuclear translocation and binding to TEAD1–4, inducing the expression of YAP/TAZ target genes such as connective tissue growth factor (*CTGF*), cysteine-rich angiogenic inducer 61 (*CYR61*), and ankyrin repeat domain-containing protein 1 (*ANKRD1*) [3, 4]. The YAP/TAZ signaling mediates intracellular signaling cascades in response to environmental changes, such as cell density and nutrient conditions, along with mechanical stimuli, such as extracellular matrix stiffness [9–13].

Among the identified upstream regulators of the Hippo pathway, the tumor suppressor Merlin, encoded by the neurofibromatosis type 2 (*NF2*) gene, is known to mediate translocation of LATS1 to the plasma membrane (PM), leading to its phosphorylation by MST1/2 and inhibition of YAP/TAZ activity [14–17]. Merlin contains the N-terminal band 4.1/ezrin/radixin/moesin (FERM) domain, which binds to LATS1; this domain also binds to the membrane lipid phosphatidylinositol 4,5-bisphosphate (PIP2), leading to Merlin activation by PIP2 [16, 18–21]. Biochemical and structural analyses have shown that Merlin adopts an inhibitory configuration through intramolecular interaction between its N-terminal FERM domain and C-terminal (CT) domain, and binding of PIP2 to the FERM domain releases the head-to-tail interaction, with the conformational change thereby inducing Merlin activation [20–22]. Notably, the co-presence of PIP2 significantly increases the binding affinity of Merlin for LATS1 [20, 23]. In addition, the PM localization of Merlin is important for its tumor suppressor function [16, 19, 24]. As PIP2 is mainly present in the PM [25, 26], PIP2 might therefore function as a lipid regulator that promotes the PM localization and/or activity of Merlin, thereby contributing to LATS1 activation.

Phosphoinositides, as phosphorylated derivatives of phosphatidylinositol, exert significant effects on various physiological membrane-associated events [27, 28]. The phosphoinositide PIP2 is a minor acidic phospholipid that critically regulates vesicle trafficking, lipid-mediated cell signaling, phagocytosis, and actin cytoskeletal rearrangement at the cell surface [26, 29, 30]. PIP2 plays such pleiotropic roles by mediating its interactions with target proteins, which can induce changes in their subcellular localization, activity, and/or conformation [25, 26, 29, 30]. In general, PIP2-interacting proteins harbor specific PIP2-binding modules, such as pleckstrin homology (PH) and FERM domains, or putative arginine/lysine-rich short motifs [26, 27]. PIP2 is mainly produced by type I phosphatidylinositol 4-phosphate 5-kinase (PIP5K) family members comprising PIP5Kα, PIP5Kβ, and PIP5K$\gamma$, which catalyze the phosphorylation of phosphatidylinositol 4-phosphate [25, 27]. Thus, the catalytic activity and expression level of PIP5K markedly affects the levels of PIP2 in the PM. Moreover, each PIP5K has different localizations and binding partners; thus, PIP5K isoforms retain distinct roles [25, 31, 32].

Alterations in PIP2 have been shown to regulate small GTPase RAP2-mediated mechano-signaling of the Hippo pathway, in which, PIP2 hydrolysis by phospholipase Cү1 (PLCү1) impairs RAP2-dependent activation of the MST1/2–LATS1/2 cascade, resulting in subsequent YAP/TAZ activation during focal adhesion formation [33]. In addition, phosphoinositides, especially PIP2, contribute to Hippo pathway activation by osmotic pressure, wherein ADP-ribosylation factor 6-dependent PIP5K activation, followed by an increase in PIP2 levels, mediates the PM recruitment of Merlin, consequently leading to LATS1 activation [34]. These findings suggest that alterations in PIP2 levels by PIP2-metabolizing enzymes, such as PLCү1 and PIP5K, are involved in the transmission of certain extracellular signals mediated by the Hippo-YAP/TAZ pathway. However, how the PIP5K-mediated PIP2 pool precisely coordinates the interaction and colocalization of Merlin with LATS1 at the PM remains unclear.

In this study, we aimed to determine the potential role of PIP5Kα, an isoform of PIP5K, in regulating the Hippo-YAP/TAZ pathway. To this end, we evaluated the effects of PIP5Kα on the phosphorylation, subcellular localization, and/or activity of LATS1, YAP, and TAZ, and on cell proliferation and stem cell differentiation. To identify relevant underlying mechanisms, we focused on the interaction and PM colocalization of PIP5Kα with Merlin and LATS1, together with their association with the lipid product PIP2. In addition, we examined PIP5Kα-mediated Hippo pathway regulation under Hippo signaling conditions, such as low/high cell density and serum starvation/stimulation, which remains largely unexplored. We provide novel evidence that PIP5Kα directly interacts with Merlin and promotes the interaction between Merlin and LATS1, facilitating their assembly and enrichment at PIP2-containing PM sites, thereby contributing to Hippo pathway activation.

## Materials and methods

### Reagents and antibodies

Most chemicals, including dexamethasone, 3-isobutyl-1-methylxanthine, insulin, saponin, cycloheximide (CHX), verteporfin, crystal violet, anti-FLAG M2 affinity gels, Dulbecco's modified Eagle medium (DMEM), and antibodies against vinculin (V4505), β-actin (A2228), α-tubulin (T5168), and FLAG-tag (F1804) were purchased from Sigma-Aldrich (St. Louis, MO, USA). Opti-MEM, Lipofectamine 2000, Lipofectamine RNAiMAX, bovine serum albumin, goat serum, 4′,6-diamidino-2-phenylindole (DAPI), and Alexa Fluor-conjugated secondary antibodies were purchased from Thermo Fisher Scientific (Waltham, MA, USA). Antibodies against PIP5Kα (#9693), PIP5Kγ (#3296), phospho-YAP (Ser127; #4911), YAP (#4912), phospho-LATS1 (Ser909; #9157), LATS1(#9153), phospho-TAZ (Ser89; #59971), TAZ (#4883), Merlin (#6995), HA-tag (#3724), Myc-tag (#2278 and #2276), p44/42 mitogen-activated protein kinase (MAPK, #4695), phospho-p44/42 MAPK (Thr202/Tyr204; #8544), p38 MAPK (#9212), phospho-p38 MAPK (Thr180/Tyr182; #9211), c-Jun N-terminal kinase (JNK, #9258), phospho-JNK (Thr183/Tyr185; #4668), Akt (#9272), and phospho-Akt (Ser473; #9271) were obtained from Cell Signaling Technology (Danvers, MA, USA). Antibodies against lamin B1 (sc-374015), GAPDH (sc-47724), and green fluorescent protein (GFP, sc-9996) were obtained from Santa Cruz Biotechnology (Dallas, TX, USA).

### Expression constructs

FLAG-, Myc-, GFP-, or monomeric red fluorescent protein (mRFP)-tagged PIP5Kα, FLAG- or GFP-tagged PIP5Kα kinase-dead (KD) mutant (D309N, R427Q), and the N-terminal (1–65 aa), catalytic (66–434 aa), CT (435–546 aa), and CT-deleted (1–434 aa; ΔCT) regions of PIP5Kα subcloned into pGEX-6P-1 and/or pcDNA3-FLAG vectors have been described previously [35, 36]. HA-tagged PIP5Kα, PIP5Kβ, or PIP5Kγ90 expression plasmids were previously provided by Dr. Michael Krauss (Leibniz-Institute for Molecular Pharmacology, Berlin, Germany) [37]. FLAG- (#11623), HA- (#32836), or GFP-tagged (#84293) Merlin, glutathione *S*-transferase (GST, #11631)- or FLAG-tagged (#11625) Merlin FERM domain, and FLAG-YAP (#66853), HA-TAZ (#32839), FLAG-TAZ (#27318), HA-NEDD4 (#27002), HA-ubiquitin (#17608), and HA-ubiquitin K48R (#17604) were obtained from Addgene (Cambridge, MA, USA). The F1 (18–98 aa), F2 (111–213 aa), and F3 (221–312 aa) regions of the Merlin FERM domain [23] were amplified from the GFP-Merlin plasmid by polymerase chain reaction (PCR) and subcloned into the EcoRI–BamHI sites of pEGFP-C2 vector. Myc-Merlin and HA-LATS1 plasmids were provided by Dr. Eunjeong Seo (OliPass Corporation, Yongin, Gyeonggi, Republic of Korea) and HA-Merlin L64P was a gift from Prof. Jung Soon Mo (Ajou University, Suwon, Republic of Korea).

### Cell culture and transfection

Human cell lines, including HEK293, HeLa, NCI-N87, SKBR3, SW480, and HFE145, and the mouse C3H10T1/2 cell line were purchased from the American Type Culture Collection (Manassas, VA, USA). Wild-type (WT), Merlin knockout (KO), and LATS1/2 KO HEK293A cells were provided by Prof. Jung Soon Mo (Ajou University). All cell lines were cultured in DMEM supplemented with 10% fetal bovine serum (FBS) and penicillin/streptomycin (HyClone, Logan, UT, USA) at 37 °C in a humidified atmosphere (5% CO_2_, 95% air) and were routinely subcultured at 2- or 3-day intervals. Cells were transiently transfected for 24 h in Opti-MEM with the indicated plasmids or corresponding empty vectors mixed with Lipofectamine 2000.

### PIP5Kα KO and gene knockdown

Control and PIP5Kα KO HeLa, HEK293, and NCI-N87 cells were generated using the CRISPR/Cas9 system as described previously [35]. Briefly, a single guide RNA (sgRNA) targeting PIP5Kα (sense, 5′-caccgCGCCCTGCCGGGCTTACCTG-3′ and antisense, 5′-aaacCAGGTAAGCCCGGCAGGGCGc-3′) or a non-targeting sgRNA (sense, 5′-caccgATCGTTTCCGCTTAACGGCG-3′ and antisense, 5′-aaacCGCCGTTAAGCGGAAACGATc-3′) was cloned into the lentiCRISPRv2 vector (Addgene #52961). Similarly, the PIP5Kα short hairpin RNA (shRNA) targeting the sequence (5′-CCATTACAATGACTTTCGATT-3′, #TRCN0000024515) or a non-targeting shRNA (5′-CAACAAGATGAAGAGCACCAA-3′) cloned into the pLKO.1 vector (Sigma-Aldrich) were used for mouse PIP5Kα knockdown, as described previously [36]. Lentiviral production, infection, and isolation of puromycin-resistant clones were carried out according to the supplier’s instructions. For knockdown of human Merlin and LATS1, double-stranded small interfering RNAs (siRNAs), 5′-UGGCCAACGAAGCACUGAUdTdT-3′ and 5′-GAACCAAACUCUCAAACAAdTdT-3′, respectively, from Bioneer (Daejeon, Republic of Korea), were mixed with Lipofectamine RNAiMAX in Opti-MEM and added to cells for 48 h. A non-targeting siRNA (5′-UUCUCCGAACGUGUCACGUdTdT-3′) was used as a negative control.

### Immunoprecipitation (IP) and western blotting (WB)

Cells were harvested in cold lysis buffer (50 mM Tris pH 7.4, 150 mM NaCl, 1 mM ethylenediaminetetraacetic acid, 1 mM egtazic acid, 1 mM dithiothreitol, 1 mM Na_3_VO_4_, 5 mM NaF, and 1% Triton X-100) containing protease and phosphatase inhibitor cocktails (GenDEPOT, Barker, TX, USA). After clearance by centrifugation (15,000 × *g*, 30 min, 4 °C), the protein concentration in the cell lysates was determined using bicinchoninic acid protein assay reagents (Pierce, Rockford, IL, USA). FLAG- and HA-tagged proteins were immunoprecipitated by mixing cell lysates (1.2–1.5 mg) with anti-FLAG M2 affinity gels (20 μl) and anti-HA antibody (2 μg), respectively, for 4–6 h at 4 °C [35, 38]. Endogenous PIP5Kα or Merlin was immunoprecipitated by incubating cell lysates (2.0 mg) with the respective specific antibody (4 μg) or normal IgG (Cell Signaling Technology) as a negative control. For IP of HA-tagged and endogenous proteins, 25 μl of protein A/G PLUS-Agarose IP reagent (Santa Cruz Biotechnology) was added for an additional 4 h. After washing five times with the cell lysis buffer, the immune complexes were analyzed by sodium dodecyl sulfate polyacrylamide gel electrophoresis (SDS-PAGE) and WB.

### Quantitative real-time reverse transcription PCR (qRT-PCR)

Total RNA was purified using the RNeasy Mini Kit (Qiagen, Hilden, Germany), and cDNA was synthesized using ReverTra Ace qPCR RT Master Mix (Toyobo, Osaka, Japan) according to the manufacturer’s instructions. qRT-PCR was performed using a QuantStudio™ 3 Real-Time PCR System (Thermo Fisher Scientific) with TOPreal™ SYBR Green qPCR 2X PreMIX (Enzynomics, Daejeon, Republic of Korea). The specific PCR primers (Additional file 1: Table S1) were obtained from Bioneer. PCR samples were prepared in triplicate, mRNA expression levels were normalized to those of *GAPDH*, and the relative expression was determined using the 2^−ΔΔCt^ method.

### Immunostaining and cell imaging

HeLa cells on an 18 mm circular coverslip coated with poly-D-lysine (Sigma-Aldrich) were fixed with 4% paraformaldehyde for 20 min, permeabilized with phosphate-buffered saline (PBS) containing 0.1% Triton X-100 for 15 min, and blocked with 10% bovine serum albumin and 5% goat serum in PBS for 30 min. The coverslips were stained with the indicated primary antibodies diluted in blocking buffer for 2 h at 25 °C, followed by staining with Alexa Fluor-conjugated secondary antibodies for 1 h. Nuclei were stained with DAPI in PBS for 5 min. For PIP2 immunostaining, the cells were permeabilized with 0.5% saponin and blocked with 10% goat serum in Tris-buffered saline. PIP2 was immunostained by sequential incubation with an anti-PIP2 mouse IgM antibody (Echelon Biosciences, Salt Lake City, UT, USA), biotinylated goat anti-mouse IgM, and Alexa Fluor-conjugated streptavidin (Thermo Fisher Scientific), according to the manufacturer’s protocol [36]. Immunofluorescence images and those of transfected GFP-, mRFP-, and yellow fluorescent protein (YFP)-tagged proteins were captured using a Zeiss LSM 710 confocal microscope (Carl Zeiss GmbH, Jena, Germany) [35, 36].

### PIP2 measurement

Cells were transfected with GFP-PLCδ-PH or Tubby-YFP expression plasmid as a PIP2 reporter, and GFP and YFP images were obtained by confocal microscopy [35]. Alternatively, cellular lipids were extracted, and PIP2 amounts were determined using a PIP2 Mass ELISA Kit (#K-4500, Echelon Biosciences) following the manufacturer’s protocol [36]. Absorbance was measured at 450 nm using a BioTek Synergy H1 Plate Reader (Agilent Technologies, Santa Clara, CA, USA). PIP2 was quantified from a standard curve fitted by four-parameter nonlinear regression.

### GST-fusion protein pull-down assay

GST-fusion proteins expressed in *Escherichia coli* strain BL21 were affinity-purified and used for pull-down assays as described previously [38]. Briefly, *E. coli* extracts prepared in PBS containing 1% Triton X-100, 1 mM dithiothreitol, and protease inhibitor cocktail were mixed with glutathione Sepharose 4 B beads (GE Healthcare, Princeton, NJ, USA) for 2 h at 4 °C. GST-fusion proteins conjugated to glutathione beads were then incubated with the cell lysates (2.0 mg) for 4 h at 4 °C. The resulting beads were washed with cell lysis buffer and analyzed using SDS-PAGE and WB.

### Subcellular fractionation

Cytoplasmic and nuclear fractions were isolated using a Nuclear Extraction Kit (#2900, Merck Millipore, Billerica, MA, USA) following the manufacturer’s instructions. Enrichment of cytosolic and nuclear proteins was evaluated using WB analysis of α-tubulin and lamin B1, respectively.

### Luciferase reporter assay

Cells were cotransfected with 1.5 μg of YAP/TAZ-responsive 8 × GTIIC-luciferase reporter plasmid (Addgene #34615) and 0.15 μg of pRL-SV40P (Addgene #27163). The luciferase assay was performed using the Dual-Luciferase Reporter Assay System (#E1910, Promega, Madison, WI, USA) according to the manufacturer’s protocol [36]. The *Renilla* luciferase reporter was used to normalize transfection efficiency. The luciferase activity was measured using a BioTek Synergy H1 Plate Reader.

### Colony formation assay

Control and PIP5Kα KO cells were seeded in 6-well plates at a density of 3,000 cells/well. PIP5Kα KO cells reconstituted with FLAG-PIP5Kα plasmids were initially plated at the same density, then repeatedly transfected with the plasmids every two days. Cells were treated with verteporfin at 2-day intervals under the indicated conditions. After one week, cells were fixed in a mixture of acetic acid:methanol (1:7) for 1 min at 25 °C, followed by staining with 0.5% crystal violet. The number of cell colonies was counted using ImageJ software (National Institutes of Health, Bethesda, MD, USA) [35].

### Adipocyte differentiation

C3H10T1/2 cells (1 × 10^5^/cm^2^) were incubated in DMEM supplemented with 5% FBS, 1 μM dexamethasone, 10 μg/ml insulin, and 0.5 mM 3-isobutyl-1-methylxanthine for two days, then the medium was replaced with DMEM containing 5% FBS and 10 μg/ml insulin [39]. After an additional 2 days, the cells were maintained in DMEM containing 5% FBS only, which was refreshed every 2 days until lipid droplets were visible through direct observation using an inverted microscope (DMi1 model, Leica Microsystems). On day 12 after the first medium exchange, the lipids droplets were stained with Oil Red O reagent (Sigma-Aldrich).

### Statistical analysis

All experiments were performed independently at least three times, with similar results. Band intensities of the western blots were measured using ImageJ software. Data shown in the graphs are presented as the mean ± S.E.M. The statistical significance (*p* values < 0.05) of the data was determined using GraphPad Prism software (La Jolla, CA, USA). An unpaired Student’s *t*-test was used to compare two groups, and one-way analysis of variance with Tukey’s multiple comparison tests was used for three or more groups.

## Results

### PIP5Kα regulates the Hippo-YAP/TAZ pathway in a catalytic activity-dependent manner

**Fig. 1:**
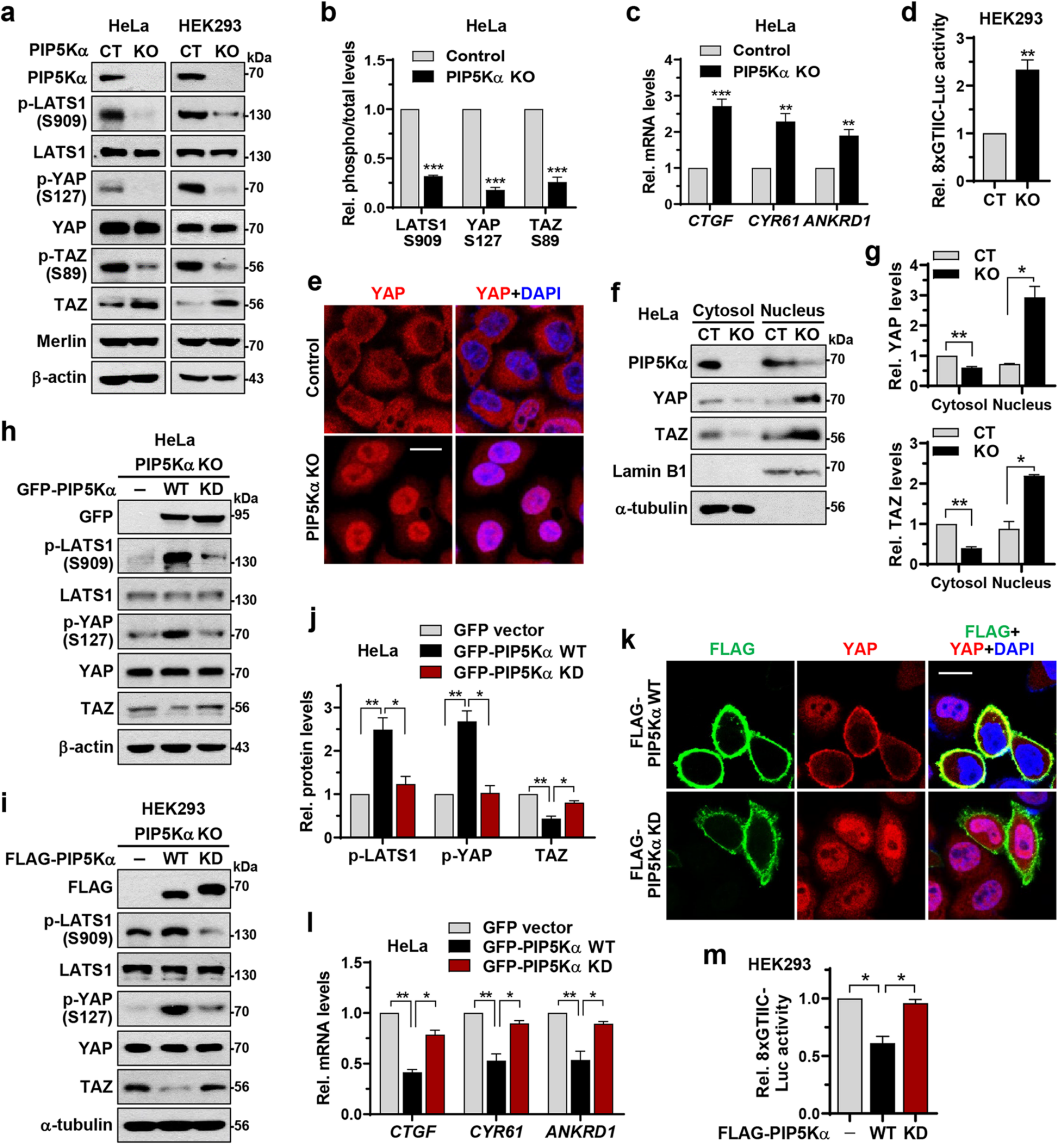
PIP5Kα activates LATS1 and inhibits YAP/TAZ in a kinase activity-dependent manner. **a** Control (CT) and PIP5Kα KO cell lysates were analyzed by WB using the indicated antibodies. **b** Relative quantification of phosphorylation/total ratios of LATS1, YAP, and TAZ in HeLa cells in (**a**) (*n* = 5). Relative quantification of YAP/TAZ target gene induction (**c**) and TEAD-binding activity (**d**) as measured using qRT-PCR (*n* = 4) and luciferase reporter assay (*n* = 3), respectively. **e** Representative confocal images of YAP immunofluorescence in control and PIP5Kα KO HeLa cells. Nuclei were visualized by DAPI staining. Scale bar, 20 μm. **f**, **g** YAP and TAZ protein levels in cytosolic and nuclear fractions from control and PIP5Kα KO HeLa cells, as analyzed by WB and quantified relative to those in the control cytosolic fraction (*n* = 4). Fractionation was confirmed by the absence and presence of lamin B1 and α-tubulin. PIP5Kα KO HeLa (**h**, **k, l**) and HEK293 (**i**,** m**) cells were reconstituted with the indicated PIP5Kα WT or KD plasmids or corresponding control vectors. **h**, **i** Cell lysates were analyzed by WB using the indicated antibodies. **j** Relative quantification of LATS1 and YAP phosphorylation and TAZ protein levels in (**h**) (*n* = 4). **k** Representative confocal images of FLAG and YAP immunofluorescence. Nuclei were stained with DAPI. Scale bar, 20 μm. YAP/TAZ target gene induction (**l**) and YAP/TAZ activity (**m**) were analyzed by qRT-PCR (*n* = 5) and luciferase reporter assay (*n* = 4), respectively, and were quantified relative to the values obtained using the control vector. Values in the graphs represent the means ± SEM. *p < 0.05, **p < 0.01

To examine the potential role of PIP5Kα in the Hippo-YAP/TAZ pathway, we evaluated changes in the phosphorylation of LATS1 (serine 909), YAP (serine 127), and TAZ (serine 89) [40–42] in PIP5Kα KO HeLa and HEK293 cells. Phosphorylation levels of all three proteins were markedly reduced in both PIP5Kα KO cell types compared with those in control cells (Fig. 1a, b). Similar decreases in LATS1 and YAP phosphorylation were also observed in PIP5Kα KO NCI-N87 cells (Additional file 1: Fig. S1a). TAZ protein levels were enhanced in PIP5Kα KO cells, whereas Merlin levels remained unchanged (Fig. 1a). Conversely, FLAG-PIP5Kα overexpression increased the phosphorylation of LATS1 and YAP, and decreased TAZ levels (Additional file 1: Fig. S1b, c). Consistent with these results, the mRNA levels of the YAP/TAZ target genes *CTGF*, *CYR61*, and *ANKRD1* [3, 43] were elevated in PIP5Kα KO HeLa cells (Fig. 1c) but were downregulated in PIP5Kα-overexpressing cells (Additional file 1: Fig. S1d). PIP5Kα KO or overexpression only minimally affected *LATS1*, *YAP*, and *TAZ* transcription (Additional file 1: Fig. S1e–g). The TEAD-binding activity of YAP/TAZ was higher in PIP5Kα KO cells than in control cells, as shown by the synthetic 8 × GTIIC-luciferase reporter assay [44] (Fig. 1d). Confocal imaging demonstrated that nuclear localization of YAP was more evident in PIP5Kα KO cells than in control cells (Fig. 1e). Cell fractionation analysis also revealed that the nuclear enrichment of YAP and TAZ was relatively high in PIP5Kα KO cells (Fig. 1f, g), but was reduced in PIP5Kα-overexpressing cells (Additional file 1: Fig. S1h). Given that LATS1-phosphorylated TAZ undergoes degradation via the ubiquitin–proteasome system (UPS) [42], we next evaluated the effects of PIP5K KO on TAZ protein stability. Chase assays using CHX, a protein synthesis inhibitor, showed that TAZ remained at relatively high levels in PIP5Kα KO cells (Additional file 1: Fig. S2a). Alternatively, PIP5Kα overexpression enhanced TAZ polyubiquitination (Additional file 1: Fig. S2b). As expected, the presence of K48R ubiquitin, which does not mediate UPS-dependent proteolysis [45], blocked the TAZ degradation induced by PIP5Kα overexpression (Additional file 1: Fig. S2c).

PIP5Kα deficiency decreased the levels of PIP2 and PIP5Kα, as revealed by immunostaining and PIP2 ELISA (Additional file 1: Fig. S3a–c). PIP2 levels were also evaluated using PIP2-specific fluorescent probes, encoded by GFP-PLCδ-PH and Tubby-YFP plasmids. The PH domain of PLCδ or Tubby has a binding affinity for PIP2; thus, the fluorescent proteins undergo differential translocation between the PM and cytoplasm depending on PIP2 levels [35, 46]. Transfected Tubby-YFP localized to the PM in control cells, but diffused into the cytoplasm in PIP5Kα KO cells (Additional file 1: Fig. S3d), indicative of a reduction in PIP2 levels upon PIP5Kα KO. As expected, GFP-PLCδ-PH and Tubby-YFP cotransfected with mRFP- and FLAG-tagged PIP5Kα WT, respectively, were concentrated in the PM; however, Tubby-YFP became highly soluble in cells cotransfected with a FLAG-PIP5Kα KD [36, 47] (Additional file 1: Fig. S3e, f). We next tested PIP5Kα WT and KD for their effects on Hippo-YAP/TAZ signaling. Reconstitution of PIP5Kα levels by transfecting GFP- and FLAG-tagged PIP5Kα WT into PIP5Kα KO HeLa and HEK293 cells, respectively, increased phosphorylation of LATS1 and YAP and decreased TAZ levels; in contrast, reconstitution with PIP5Kα KD did not strongly induce such changes (Fig. 1h–j). The nuclear localization of YAP was more pronounced in FLAG-PIP5Kα KD-reconstituted cells than in FLAG-PIP5Kα WT-reconstituted cells (Fig. 1k). Consistent with this, GFP-PIP5Kα KD reconstitution resulted in relatively high *CTGF*, *CYR61*, and *ANKRD1* transcription and TEAD-binding YAP/TAZ activity compared with the results following PIP5Kα WT reconstitution (Fig. 1l, m). Similarly, overexpression of FLAG-PIP5Kα KD resulted in relatively lower YAP phosphorylation in SKBR3 and HFE145 cells (Additional file 1: Fig. S4a, b), and higher *CTGF* and *CYR61* transcription in SKBR3 and NCI-N87 cells (Additional file 1: Fig. S4c, d) than the results obtained upon WT overexpression. In addition, FLAG-YAP or HA-TAZ cotransfected with GFP-PIP5Kα KD localized primarily to the nucleus, in contrast to the localization upon GFP-PIP5Kα WT cotransfection (Additional file 1: Fig. S4e, f).

As controls, we tested PIP5Kα KO and KD mutant for their potential effects on PIP5Kβ and PIP5Kγ expression. PIP5Kα KO or its KD mutant did not show substantial alterations in PIP5Kγ protein and mRNA levels and PIP5Kβ mRNA levels in HeLa and HEK293 cells (Additional file 1: Fig. S5). We then tested whether PIP5Kβ and/or PIP5Kγ may also play a role in the Hippo-YAP/TAZ pathway using ectopic expression. Transfected HA-PIP5Kβ or HA-PIP5Kγ90 (a PIP5Kγ splice variant having 90 kDa molecular weight) induced increases in LATS1 and YAP phosphorylations although their effects were slightly lower than those of HA-PIP5Kα (Additional file 1: Fig. S6a, b). Similarly, overexpression of the three HA-PIP5K isoforms downregulated YAP/TAZ target gene induction at comparable levels (Additional file 1: Fig. S6c). As another controls, we further addressed whether PIP5Kα KO might affect other signaling pathways by examining its potential effects on phosphorylation changes in MAPK family proteins and Akt. Phosphorylation levels of p44/42 MAPK and JNK were not altered in PIP5Kα KO HeLa and HEK293 cells compared with those in control cells (Additional file 1: Fig. S7). p38 MAPK phosphorylation levels were increased in both PIP5Kα KO cells to some extent while Akt phosphorylation levels were modestly decreased in PIP5Kα KO HeLa cells but not in PIP5Kα KO HEK293 cells (Additional file 1: Fig. S7).

### PIP5Kα plays a role in cell density- and serum stimulation-dependent Hippo signaling

**Fig. 2:**
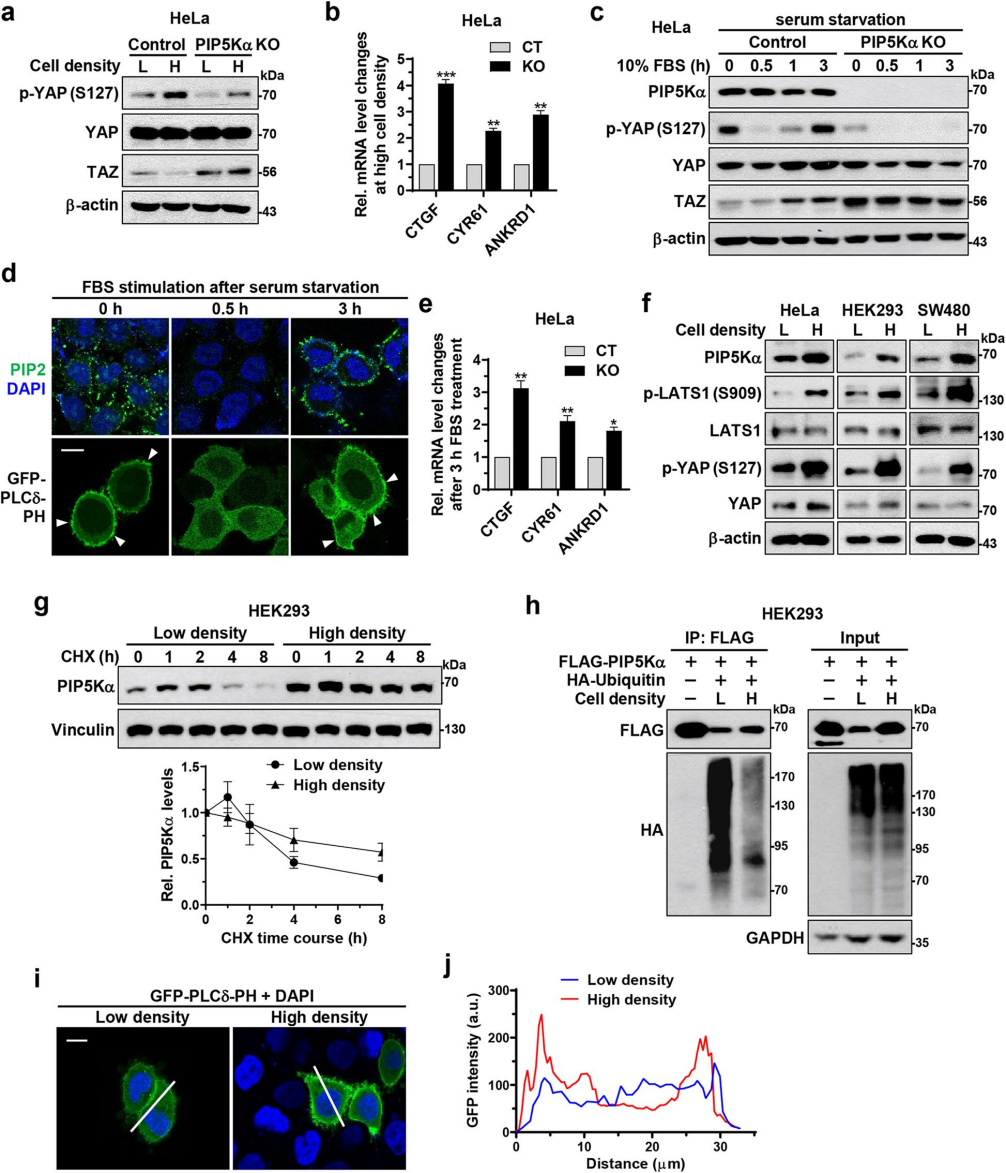
PIP5Kα participates in Hippo signaling related to cell density and serum stimulation. Control and PIP5Kα KO HeLa cells (**a**-**c**,** e**) or WT HeLa cells (**d**) were evaluated. **a** WB analysis of cell lysates prepared at low (L, 4 × 10^4^/cm^2^) and high (H, 2 × 10^5^/cm^2^) density. Relative quantification of YAP/TAZ target gene induction at high cell density (**b**) (*n* = 4) or after serum stimulation (**e**) (*n* = 4). Values in the graphs represent the means ± SEM. *p < 0.05, **p < 0.01, ***p < 0.001. Cells were serum-starved overnight and then stimulated with 10% FBS for the indicated times (**c**, **d**) or 3 h (**e**). **c** Cell lysates were analyzed by WB with the indicated antibodies. **d** Representative confocal images of PIP2 immunofluorescence and DAPI staining (top) and transfected GFP-PLCδ-PH (bottom). The arrowheads indicate the presence of PIP2 at the PM. Scale bar, 10 μm. **f** WB analysis of HeLa, HEK293, and SW480 cell lysates at low and high density with the indicated antibodies. **g** Cell density-dependent PIP5Kα protein stability ascertained using the CHX (100 μM) chase assay, and relative quantification of PIP5Kα levels at a respective zero-time point (*n* = 4). Vinculin immunoblotting was included as a loading control. Values in the graphs represent the means ± SEM. **h** WB analysis of cell lysates (input) and FLAG-IP products at low and high density following cotransfection of FLAG-PIP5Kα and HA-ubiquitin. **i** Representative confocal images of transfected with GFP-PLCδ-PH fluorescence in HeLa cells at low and high density. Cells were outlined by DAPI staining. Scale bar, 10 μm. **j** GFP intensity profiles along the lines in (**i**) acquired using Zeiss ZEN imaging software

Environmental cues such as high cell density or serum starvation activate the Hippo pathway, leading to YAP/TAZ inhibition, whereas low cell density or serum stimulation has the opposite effect [10–12]. Here, we examined the regulatory role of PIP5Kα in Hippo signaling. YAP and TAZ in control cells underwent phosphorylation and downregulation, respectively, as cell confluence increased, whereas these changes became blunted in PIP5Kα KO cells (Fig. 2a, Additional file 1: Fig. S8a, b). At high cell densities, *CTGF*, *CYR61*, and *ANKRD1* mRNA levels were relatively higher in PIP5Kα KO cells than in control cells (Fig. 2b). Upon serum stimulation following starvation, YAP phosphorylation in control cells declined rapidly within 30 min, followed by gradual recovery during the 3 h period, similar to the previously reported results [41]; however, it remained at very low levels in PIP5Kα KO cells (Fig. 2c, Additional file 1: Fig. S8c). In accordance with the YAP phosphorylation changes, PIP2 immunostaining and GFP-PLCδ-PH imaging demonstrated an initial decrease followed by a rise in PIP2 levels at 30 min and 3 h, respectively, in response to serum stimulation (Fig. 2d). TAZ protein levels remained relatively high in PIP5Kα KO cells throughout the 3 h period (Fig. 2c, Additional file 1: Fig. S8d). After serum stimulation for 3 h, *CTGF*, *CYR61*, and *ANKRD1* mRNA levels were higher in PIP5Kα KO cells than in the control cells (Fig. 2e).

Notably, PIP5Kα protein levels were upregulated at high cell density compared to those at low cell density in HeLa, HEK293, and SW480 cells, whereas its mRNA levels were not altered (Fig. 2f, Additional file 1: Fig. S8e). Under high-density conditions, phosphorylation of LATS1 and YAP increased, whereas *CTGF* and *CYR61* mRNA levels decreased, as expected (Fig. 2f, Additional file 1: Fig. S8f). We previously found that PIP5Kα is a substrate for UPS-mediated degradation [35]. Therefore, we tested whether cell density could affect PIP5Kα protein stability. Reduction in PIP5Kα protein levels was detectable 4–8 h following CHX treatment at low cell density, but was slowed down at high cell density throughout the 8 h period (Fig. 2g). Moreover, cotransfection with FLAG-PIP5Kα and HA-ubiquitin resulted in much lower PIP5Kα ubiquitination levels at high cell density than those observed at low cell density (Fig. 2h). In agreement with this, dense cells displayed higher PIP2 levels than sparse cells, as visualized using GFP-PLCδ-PH imaging (Fig. 2i, j).

### PIP5Kα mediates PM colocalization of LATS1 with Merlin in a PIP2-dependent manner

**Fig. 3:**
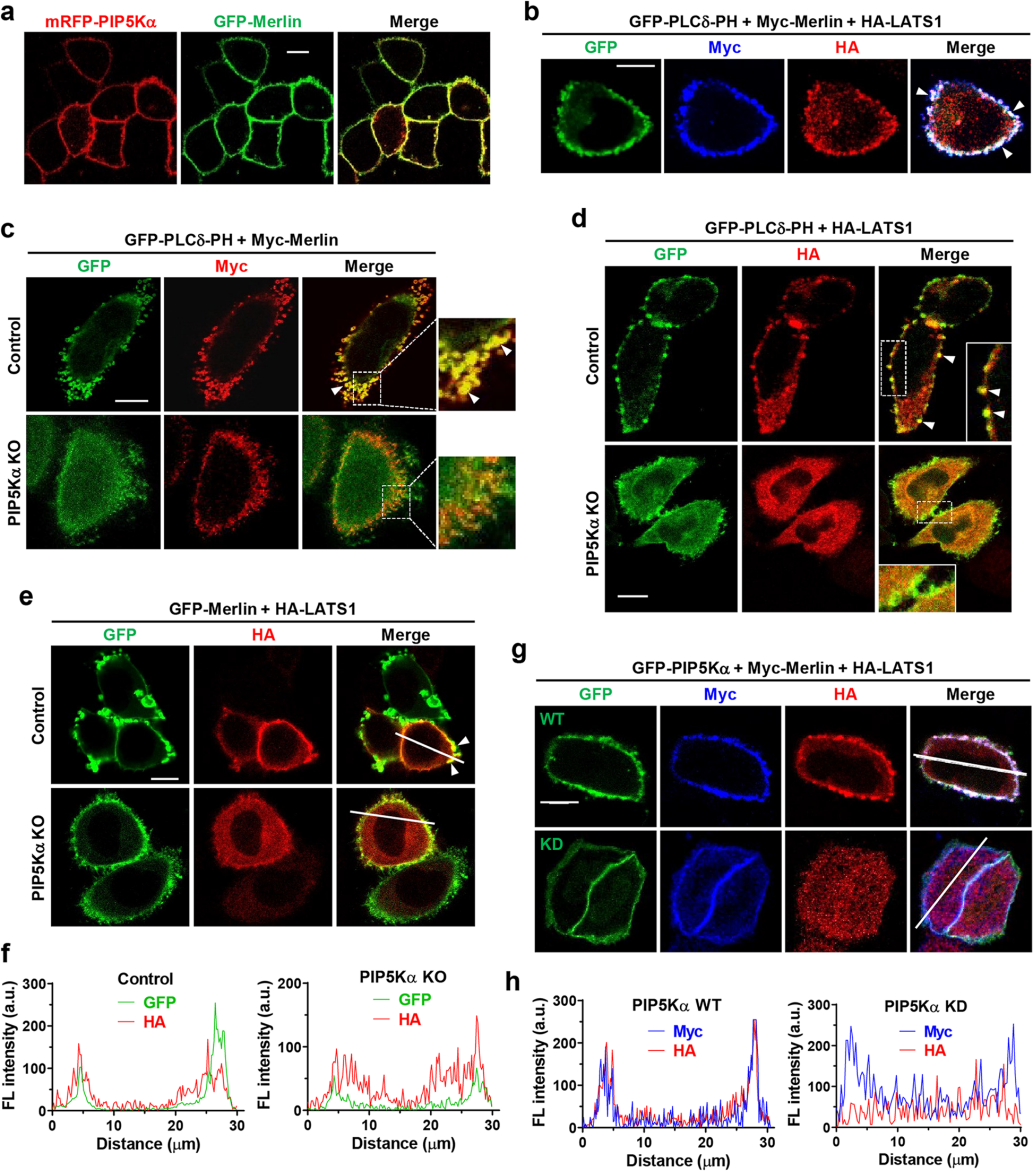
PIP5Kα-dependent PIP2 pool mediates PM colocalization between Merlin and LATS1. WT (**a**, **b**, **g**) or control and PIP5Kα KO (**c**-**e**) -HeLa cells were cotransfected with the indicated plasmids and representative images were obtained by confocal microscopy. **a** Colocalization of mRFP-PIP5Kα with GFP-Merlin at the PM. **b**-**d** PIP2 levels were monitored via its fluorescent reporter GFP-PLCδ-PH. **b**-**e**, **g** Myc-Merlin and HA-LATS1 were visualized using Myc and HA immunostaining, followed by staining with Alexa Fluor 350- and/or 594-labeled secondary antibodies, respectively. **b** The arrowheads indicate colocalization of Myc-Merlin and HA-LATS1 with PIP2. **c**,** d** The arrowheads and those in in the magnified area highlight enrichment of Myc-Merlin and HA-LATS1 at the PIP2-rich PM sites. **e** Localization of GFP-Merlin and HA-LATS1 in control and PIP5Kα KO cells. The arrowheads indicate PM colocalization of HA-LATS1 with GFP-Merlin. **f** GFP and HA fluorescent (FL) intensity profiles along the lines in (**e**). **g** Localization of Myc-Merlin and HA-LATS1 in GFP-PIP5Kα WT- or KD-cotransfected cells. **h** Myc and HA fluorescent (FL) intensity profiles along the lines in (**g**). **f**, **h** Profile graphs acquired using Zeiss ZEN imaging software. Scale bars, 10 μm

Previous studies showed that Merlin mediates recruitment of LATS1 to the PM [16] and localizes to the PM through PIP2 binding [19, 34]. As PIP5Kα and PIP2 were primarily localized to the PM (Additional file 1: Fig. S3a, b), we investigated the potential role of PIP5Kα-dependent PIP2 in modulating Merlin and LATS1 localization using cotransfection and confocal imaging. GFP-Merlin and mRFP-PIP5Kα showed robust colocalization in the PM (Fig. 3a). Myc-Merlin and HA-LATS1 colocalized at the PIP2-rich PM sites that were visualized using the PIP2 reporter GFP-PLCδ-PH (Fig. 3b). In PIP5Kα KO cells, PM localization of Myc-Merlin could be observed, however, its specific localization to the PIP2-containing PM was reduced compared with that in the control cells (Fig. 3c). HA-LATS1 localization to the PIP2-rich PM was clearly detectable in control cells but was much less prominent in PIP5Kα KO cells (Fig. 3d). Similarly, HA-LATS1 colocalized with GFP-Merlin at the PM in control cells, but distributed more widely in the cytoplasm in PIP5Kα KO cells (Fig. 3e, f). Furthermore, HA-LATS1 colocalized with GFP-PIP5Kα WT and Myc-Merlin in the PM (Fig. 3g, h). In contrast, in GFP-PIP5Kα KD-expressing cells with reduced PIP2 levels (Additional file 1: Fig. S3f), HA-LATS1 signals were markedly distinct from the Myc-Merlin-associated PM (Fig. 3g, h), similar to the results obtained in PIP5Kα KO cells. Moreover, HA-LATS1 lost its PM colocalization with mRFP-PIP5Kα and GFP-Merlin 30 min following serum stimulation, accompanied by a decrease in PIP2 levels (Fig. 2d, Additional file 1: Fig. S9a–c).

### PIP5Kα directly interacts with Merlin and promotes Merlin interaction with LATS1

**Fig. 4:**
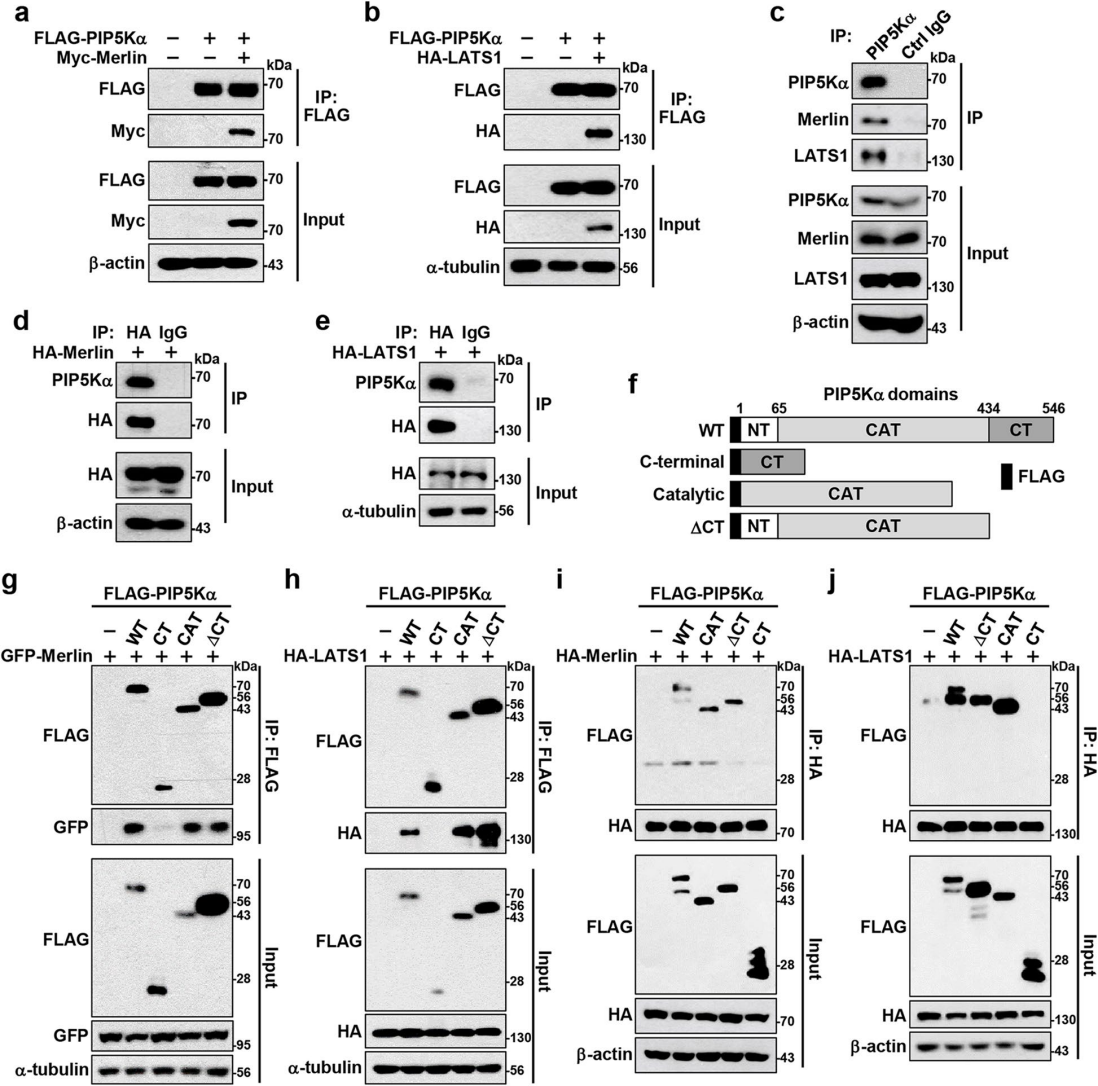
PIP5Kα coprecipitates with Merlin and LATS1 through its catalytic domain. FLAG-IP products were prepared from HEK293 cells transfected with FLAG-PIP5Kα and/or Myc-Merlin (**a**) or HA-LATS1 (**b**), as indicated. **c** HEK293 cell lysates were immunoprecipitated using an anti-PIP5Kα antibody or control IgG. HEK293 cell lysates following transfection with HA-Merlin (**d**) and HA-LATS1 (**e**) were immunoprecipitated with an anti-HA antibody or control IgG. **f** Schematic of the PIP5Kα domain structure comprising N-terminal (NT), catalytic (CAT), and CT domains and the ΔCT form. FLAG-tagged PIP5Kα WT and its truncated forms were cotransfected into HEK293 cells together with GFP-Merlin (**g**), HA-LATS1 (**h**, **j**), or HA-Merlin (**i**), as indicated. Cell lysates were then processed for FLAG IP (**g**, **h**) or HA IP (**i**, **j**). **a**-**e**, **g**-**j** Cell lysates (input) and resulting IP products were analyzed by WB using the indicated antibodies

The colocalization of PIP5Kα with Merlin and LATS1 prompted us to examine the possible protein interactions among them. Myc-Merlin or HA-LATS1 coprecipitated with FLAG-PIP5Kα upon cotransfection (Fig. 4a, b). Endogenous PIP5Kα also coprecipitated with Merlin and LATS1 (Fig. 4c). Similarly, HA-IP products from HA-Merlin or HA-LATS1 transfection contained endogenous PIP5Kα (Fig. 4d, e). To search for a relevant motif of PIP5Kα that interacted with Merlin and LATS1, FLAG-tagged truncated forms of PIP5Kα were used (Fig. 4f). The catalytic domain and ΔCT form, as well as WT, coprecipitated with cotransfected GFP-Merlin or HA-LATS1, whereas the CT domain did not (Fig. 4g, h). We obtained similar results from HA-IP products following cotransfection with the FLAG-PIP5Kα forms and HA-Merlin or HA-LATS1 (Fig. 4i, j). Alternatively, we performed pull-down assays using GST-fusion proteins of the PIP5Kα N-terminal or CT domain. Neither pulled down HA-Merlin or HA-LATS1, whereas the CT domain pulled down HA-NEDD4, as reported previously [35] (Additional file 1: Fig. S10a–c). In accordance with these results, the catalytic domain and ΔCT form, but not the CT domain, colocalized with GFP-Merlin and HA-LATS1 (Additional file 1: Fig. S11a, b).

**Fig. 5:**
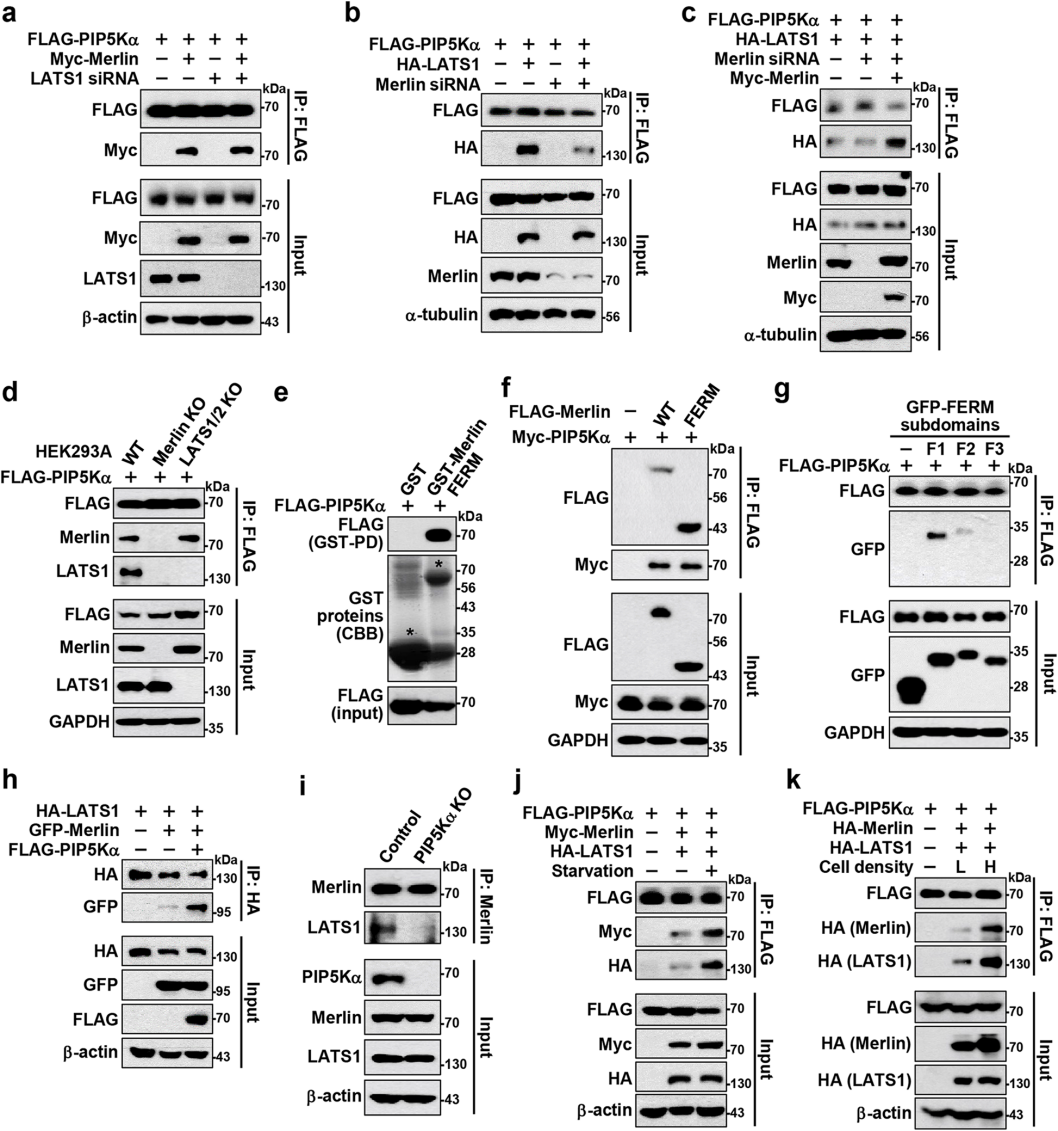
PIP5Kα interacts with the Merlin FERM domain and positively mediates interaction of Merlin with LATS1. **a**-**h**, **j**, **k** IP products and pull-down samples were obtained from transfected cell lysates prepared under the indicated conditions. **a**, **b** FLAG-PIP5Kα was cotransfected into HeLa cells in the absence and presence of Myc-Merlin or HA-LATS1 one day after treatment with control, LATS1, or Merlin siRNA, as indicated. **c** Myc-Merlin was added back to the Merlin siRNA-treated cells in (**b**). **d** FLAG-PIP5Kα was transfected into WT, Merlin KO, and LATS1/2 KO HEK293A cells. **e** GST-fusion protein pull-down (PD) assay with FLAG-PIP5Kα-transfected HEK293 cell lysates. The asterisk indicates GST and GST-Merlin FERM proteins as detected by Coomassie brilliant blue (CBB) staining. **f** HEK293 cells were cotransfected with Myc-PIP5Kα and FLAG-Merlin WT or FERM domain. **g** HEK293 cells were cotransfected with FLAG-PIP5Kα and the GFP-tagged F1, F2, or F3 subdomain of the Merlin FERM domain. **h** HA-LATS1 was cotransfected into HEK293 cells with or without GFP-Merlin and FLAG-PIP5Kα. **i** Control and PIP5Kα KO HEK293 cell lysates were immunoprecipitated with an anti-Merlin antibody. HEK293 cells cotransfected with FLAG-PIP5Kα, HA-LATS1, and Myc- or HA-Merlin, as indicated, were maintained in normal growth medium or serum starved overnight (**j**), or were harvested at low (L, 4 × 10^4^/cm^2^) and high (H, 2 × 10^5^/cm^2^) density 1 day after cell plating (**k**). **a**-**k** Cell lysates, IP products, and pull-down samples were analyzed by WB using the indicated antibodies

We then examined the details of the interactions among PIP5Kα, Merlin, and LATS1. FLAG-PIP5Kα interacted with Myc-Merlin upon LATS1 knockdown by siRNA, comparable to the results following control knockdown (Fig. 5a). In contrast, siRNA-mediated Merlin knockdown markedly reduced the interaction between FLAG-PIP5Kα and HA-LATS1 (Fig. 5b), which, in turn, was restored upon reconstitution with Myc-Merlin (Fig. 5c). Consistent with this, FLAG-PIP5Kα strongly interacted with endogenous Merlin in LATS1/2 KO cells; however, FLAG-PIP5Kα interaction with endogenous LATS1 was not apparent in Merlin KO cells (Fig. 5d). As Merlin binds to LATS1 through its FERM domain and PIP2 can affect Merlin–LATS1 interaction [16, 18, 20, 23], we next examined whether the PIP2-binding FERM domain in Merlin could interact with PIP5Kα. The GST-fusion protein of the Merlin FERM domain bound to FLAG-PIP5Kα (Fig. 5e), and the FLAG-Merlin FERM domain showed strong binding affinity for Myc-PIP5Kα (Fig. 5f). Confocal images also demonstrated strong PM colocalization between mRFP-PIP5Kα and FLAG-Merlin FERM domain (Additional file 1: Fig. S11c). The Merlin FERM domain comprises F1, F2, and F3 subdomains [18, 20]. Upon cotransfection with FLAG-PIP5Kα and the GFP-tagged subdomains, PIP5Kα coprecipitated mainly with the F1 subdomain (Fig. 5g).

We further observed that FLAG-PIP5Kα augmented the interaction between GFP-Merlin and HA-LATS1 (Fig. 5h). Conversely, PIP5Kα deficiency markedly attenuated the endogenous Merlin–LATS1 interaction (Fig. 5i). In addition, interactions of FLAG-PIP5Kα with cotransfected Merlin and LATS1 increased considerably upon serum starvation (Fig. 5j) and, similarly, at high cell density compared with low cell density (Fig. 5k). Cotransfection experiments were then performed to evaluate whether a loss-of-function mutation of Merlin (L64P) found in the inherited NF2-related diseases [48, 49] might exhibit altered interactions with PIP5Kα and LATS1. Notably, in sharp contrast to HA-Merlin WT, HA-Merlin L64P interacted only minimally with FLAG-PIP5Kα, and showed weakened binding to HA-LATS1 (Additional file 1: Fig. S12a). Moreover, the Merlin mutant was distributed in the cytoplasm and thus did not colocalize with mRFP-PIP5Kα in the PM, unlike its WT form (Additional file 1: Fig. S12b).

### PIP5Kα regulation of the Hippo pathway requires Merlin and LATS1

**Fig. 6:**
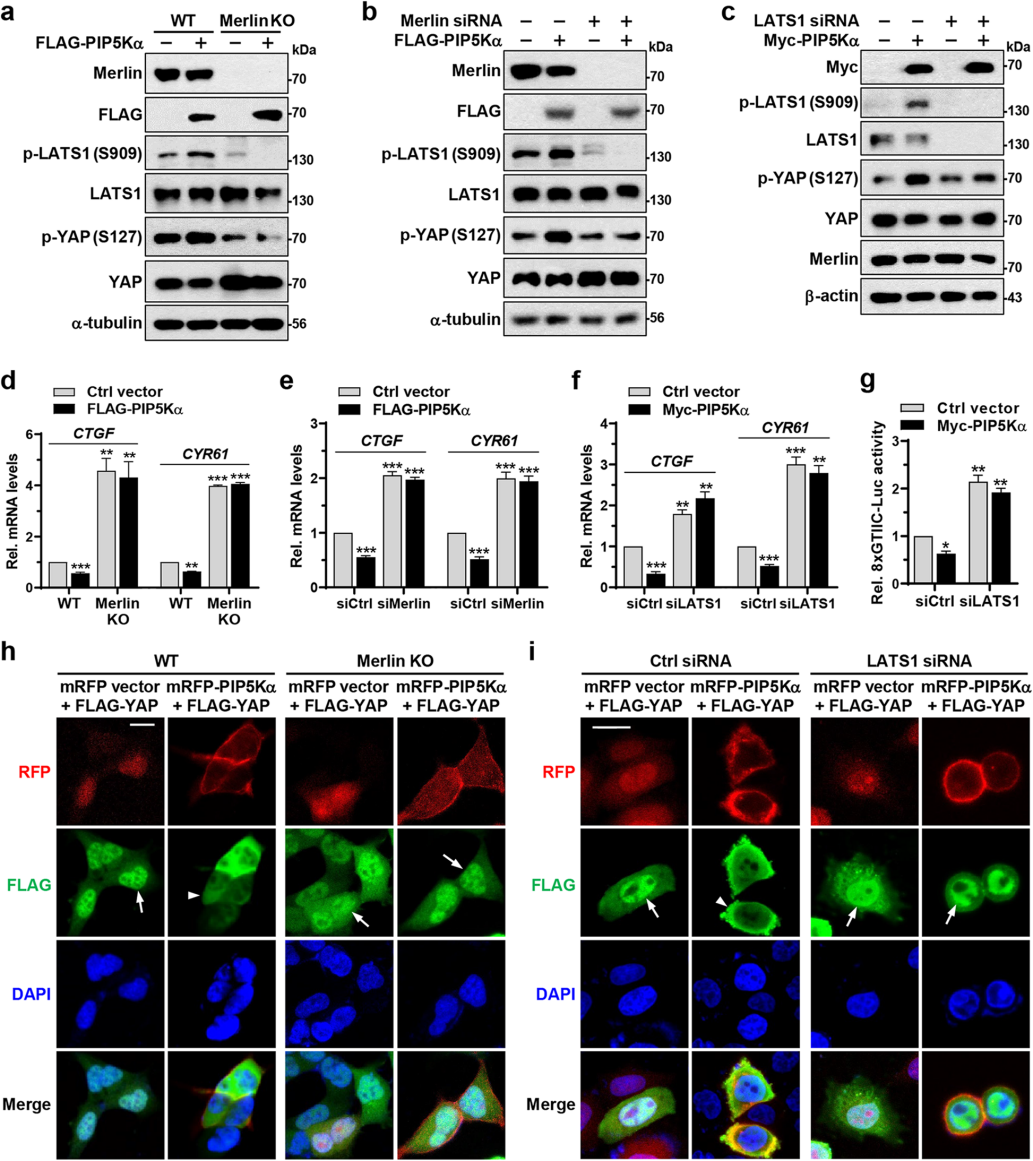
Ablation of Merlin and LATS1 compromises the regulatory effects of PIP5Kα on the Hippo pathway. **a** WT and Merlin KO HEK293A cells were transfected with the control vector or FLAG-PIP5Kα. **b**, **c** HeLa cells pretreated with control, Merlin, or LATS1 siRNA were transfected with the control vector and FLAG- or Myc-PIP5Kα, as indicated. **a**-**c** The resulting cell lysates were analyzed by WB using the indicated antibodies. **d**-**f** qRT-PCR analysis of *CTGF* and *CYR61* mRNA levels under the same conditions as (**a**-**c**), respectively (*n* = 4). **g** Measurement of YAP/TAZ activity by luciferase reporter assay under the same condition as (**c**) (*n* = 3). **d**-**g** The results were quantified relative to those obtained using the control vector and/or siRNA transfection. Values in the graphs represent the means ± SEM. *p < 0.05, **p < 0.01, ***p < 0.001. WT and Merlin KO HEK293A cells (**h**) and HeLa cells pretreated with control or LATS1 siRNA (**i**) were cotransfected with FLAG-YAP and mRFP control vector or mRFP-PIP5Kα, as indicated. Representative images of RFP, FLAG immunofluorescence, and DAPI were obtained by confocal microscopy. The arrows and arrowheads indicate the presence and absence of nuclear FLAG-YAP in the RFP-positive cells, respectively. Scale bars, 20 μm

We next examined whether the regulatory effects of PIP5Kα on the Hippo pathway are dependent on Merlin and LATS1. Merlin deficiency abolished the stimulatory effects of FLAG-PIP5Kα overexpression on LATS1 and YAP phosphorylation and lowered their basal phosphorylation levels (Fig. 6a, Additional file 1: Fig. S13a). Similarly, siRNA-mediated knockdown of Merlin (Fig. 6b, Additional file 1: Fig. S13b) and LATS1 (Fig. 6c, Additional file 1: Fig. S13c) significantly prevented the increase in LATS1 and/or YAP phosphorylation by FLAG- and Myc-PIP5Kα overexpression, respectively. In accordance with these results, *CTGF* and *CYR61* mRNA levels were relatively high in Merlin KO (Fig. 6d) and knockdown (Fig. 6e) cells compared with those in the corresponding control cells, irrespective of PIP5Kα overexpression. Similarly, LATS1 knockdown abrogated the inhibitory effects of Myc-PIP5Kα overexpression on *CTGF* and *CYR61* transcription (Fig. 6f) and TEAD-binding YAP/TAZ activity (Fig. 6g). Comparison of the effects of PIP5Kα on YAP localization in WT and Merlin KO cells revealed that in mRFP-PIP5Kα-cotransfected cells, FLAG-YAP was highly enriched within the nucleus in Merlin KO cells, in contrast to its cytoplasmic location in WT cells (Fig. 6h). Control mRFP vector had little effect on the nuclear localization of FLAG-YAP in either cell type. Similarly, mRFP-PIP5Kα expression induced the cytosolic localization of FLAG-YAP in control cells, whereas this translocation did not occur in LATS1 knockdown cells (Fig. 6i).

### PIP5Kα regulates YAP/TAZ-mediated cell proliferation and differentiation

**Fig. 7:**
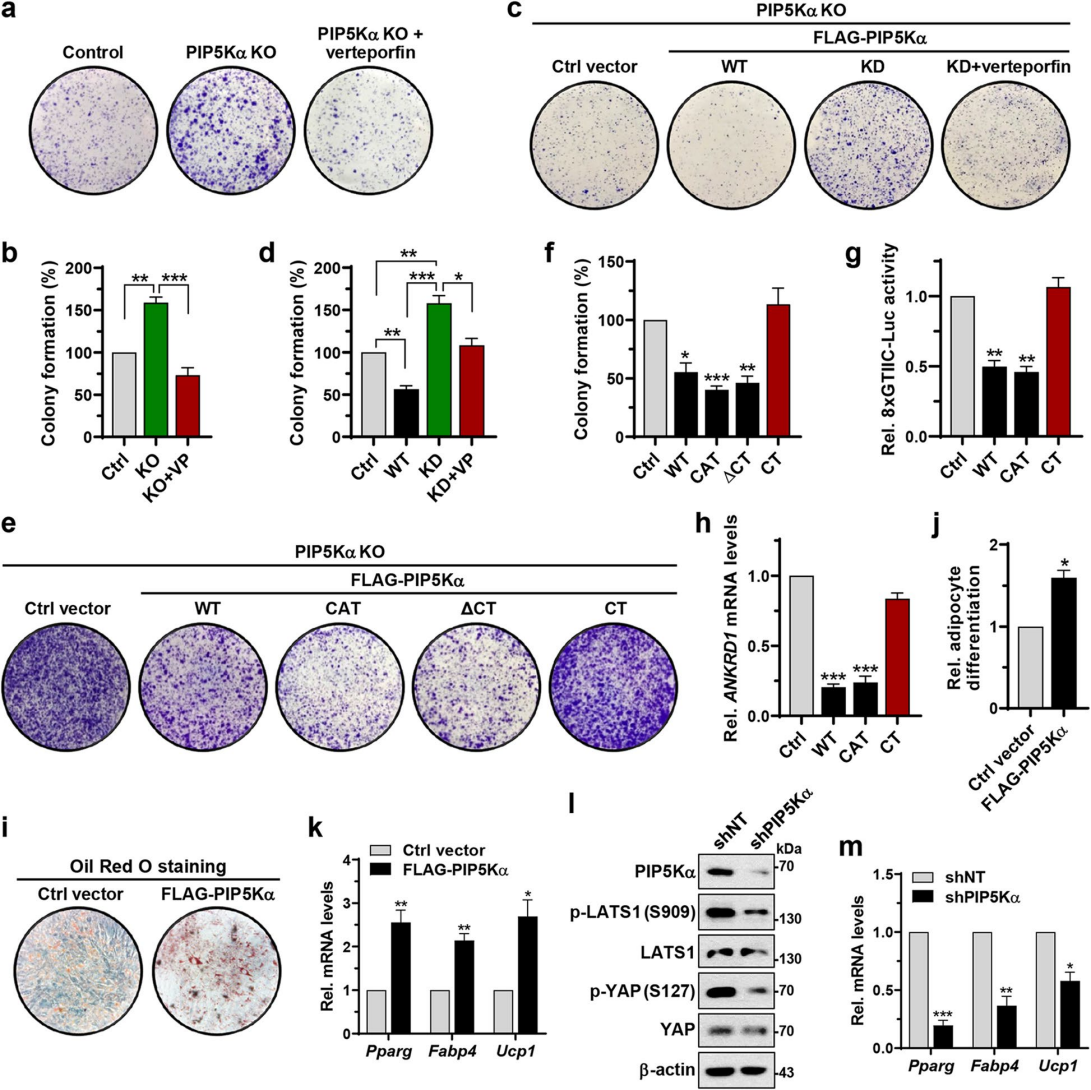
PIP5Kα plays a role in YAP/TAZ-dependent cell proliferation and differentiation. **a**, **b** Colony formation assay with control and PIP5Kα KO HeLa cells in the absence and presence of verteporfin (VP, 3 μM) and relative quantification compared with the results of the control (*n* = 4). **c**, **d** Colony formation assay with FLAG-PIP5Kα WT- or KD-reconstituted PIP5Kα KO HEK293 cells and relative quantification compared with the results of control vector reconstitution (*n* = 4). The effect of verteporfin (VP, 3 μM) on KD-reconstituted cells was included. **e**, **f** Colony formation assay with PIP5Kα KO HEK293 cells transfected with FLAG-PIP5Kα WT or its truncated forms including the catalytic (CAT) domain, CT domain, and ΔCT form and relative quantification compared with the results of control vector transfection (*n* = 3). YAP/TAZ activity (**g**) and *ANKRD1* mRNA levels (**h**) as analyzed by luciferase reporter assay (*n* = 4) and qRT-PCR (*n* = 3), respectively, under the similar condition as in (**e**). C3H10T1/2 cells transfected with the control vector or FLAG-PIP5Kα were assayed for adipogenic differentiation with Oil Red O staining (**i**, **j**) or analyzed for changes in mRNA expression levels of *Pparg*, *Fabp4*, and *Ucp1* by qRT-PCR (**k**). C3H10T1/2 cells expressing non-targeting shRNA (shNT) or PIP5Kα shRNA (shPIP5Kα) were immunoblotted using the indicated antibodies (**l**) or analyzed for *Pparg*, *Fabp4*, and *Ucp1* mRNA expression by qRT-PCR (**m**). **j**, **k**, **m** The results were quantified relative to those obtained with control vector or non-targeting shRNA (*n* = 3). Values in the graphs represent the means ± SEM. *p < 0.05, **p < 0.01, ***p < 0.001

To address the functional relevance of regulation of the Hippo pathway by PIP5Kα, we examined its effects on cell proliferation using a colony formation assay. PIP5Kα deficiency led to a marked increase in colony formation, which was antagonized by verteporfin, a chemical to disrupt the interaction of YAP/TAZ with TEAD [50] (Fig. 7a, b). Reconstitution of PIP5Kα KO cells with FLAG-PIP5Kα WT and KD oppositely regulated colony formation; the presence of verteporfin suppressed the colony formation enhanced by FLAG-PIP5Kα KD reconstitution (Fig. 7c, d). Reconstitution with the catalytic domain or ΔCT form of FLAG-PIP5Kα, which interacted with Merlin and LATS1 (Fig. 4g–j), significantly reduced colony formation, similar to the effects of FLAG-PIP5Kα WT, whereas the CT domain did not (Fig. 7e, f). Similarly, the catalytic domain, but not the CT domain, decreased TEAD-binding YAP/TAZ activity and *ANKRD1* mRNA levels to the same extent as the WT (Fig. 7g, h). Moreover, as YAP/TAZ activation inhibits the adipocyte differentiation of mesenchymal stem cells (MSCs) [3, 51, 52], we tested the effects of PIP5Kα on adipocyte differentiation in murine MSC-like C3H10T1/2 cells. FLAG-PIP5Kα overexpression yielded an adipocyte phonotype, as shown by Oil Red O staining (Fig. 7i, j), and upregulated the gene expression of peroxisome proliferator-activated receptor gamma (*Pparg*), fatty acid-binding protein 4 (*Fabp4*), and mitochondrial uncoupling protein 1 (*Ucp1*), which are markers of adipocyte differentiation [53] (Fig. 7k). As expected, shRNA-mediated PIP5Kα knockdown reduced LATS1 and YAP phosphorylation (Fig. 7l) and downregulated *Pparg*, *Fabp4*, and *Ucp1* gene expression (Fig. 7m).

## Discussion

In this study, we revealed the potent role of lipid kinase PIP5Kα in the Hippo-YAP/TAZ pathway in various cell line models. Our results showed that PIP5Kα induced stimulatory LATS1 phosphorylation, inhibitory phosphorylation of YAP and TAZ, and TAZ degradation. Consistent with this, PIP5Kα repressed the nuclear enrichment of YAP/TAZ and induction of their target genes. In contrast, a catalytically inactive PIP5Kα mutant did not exert such effects, indicating that the regulatory effects of PIP5Kα on the Hippo-YAP/TAZ pathway are dependent on its lipid product PIP2. Both Merlin deficiency and LATS1 knockdown significantly compromised PIP5Kα-induced YAP/TAZ inactivation, confirming that Merlin and LATS1 are major downstream effectors mediating PIP5Kα-driven activation of the Hippo pathway. PIP2 is a signaling lipid that binds to and activates Merlin, thereby promoting LATS1 activation [16, 19, 20, 34]. Mechanistically, our results support a direct interaction between PIP5Kα and Merlin, which induces PIP2-dependent Merlin activation. This further mediates the recruitment of LATS1 to the PIP2-rich sites in the PM, and thus promotes the interaction of Merlin with LATS1, thereby constituting an upstream signaling machinery for Hippo pathway activation (Additional file 1: Fig. S14).

PIP2 is highly abundant in the PM relative to other organelle membranes and acts as an anchor for the translocation of cytoplasmic PIP2-binding proteins to the PM, which can also affect protein–protein interactions [26, 29, 54]. Therefore, PIP2 is a minor lipid but significantly affects various membrane events, including lipid-mediated signaling at the cell surface [25, 26, 29–31]. Merlin undergoes a conformational change from the inactive closed form to the active open form upon PIP2 binding to its FERM domain [20–23]. Thus, the binding of PIP2 to Merlin markedly promoted its interaction with LATS1; conversely, the PIP2 binding-deficient Merlin mutation significantly lowers the binding affinity for LATS1 [20, 23]. Accordingly, the Merlin mutant was much less effective in inhibiting cell proliferation-related activation of YAP and Rac1 than the WT form [20]. The association of Merlin and LATS1 with the PM is a significant feature in Hippo pathway activation [16, 40, 55]. Here, we found that both Merlin and LATS1 localized mainly to the PIP2-rich PM and colocalized with PIP5Kα at the PM. Notably, PIP5Kα deficiency or its KD mutant, which caused a reduction in PIP2 levels, diminished the PM localization of LATS1. Similarly, LATS1 dislocated from the PM when PIP2 levels were reduced by serum stimulation. Unlike LATS1, PM localization of Merlin was largely unaffected under PIP2-reduced conditions, although it did not appear to be preferentially located at PIP2-containing PM sites. This suggests that PIP5Kα-dependent PIP2 production mediates the recruitment of LATS1 to the PIP2-rich PM through promoting Merlin activation but is not an exclusive factor determining the PM localization of Merlin. Consistent with this, it has been shown that membrane proteins, such as CD44, E-cadherin, angiomotin, and integrin β1, are involved in the membrane targeting of Merlin [18, 56–59]. Thus, it is possible that these membrane proteins participate in Merlin localization to the PM, whereas PIP2 produced by PIP5Kα serves as a potent activator of Merlin.

In this study, we demonstrated not only PM colocalization but also protein interactions among PIP5Kα, Merlin, and LATS1. We found that the PIP5Kα catalytic domain interacted with the Merlin FERM domain. Merlin deficiency significantly abrogated the PIP5Kα–LATS1 interaction, whereas PIP5Kα–Merlin interaction was unaffected by LATS1 deficiency. Thus, it is likely that Merlin directly interacts with PIP5Kα but indirectly mediates the PIP5Kα–LATS1 interaction to form a ternary protein complex of PIP5Kα, Merlin, and LATS1. Notably, the Merlin–LATS1 interaction was enhanced by PIP5Kα but weakened by PIP5Kα deficiency. Based on the direct lipid–protein and protein–protein interactions of the Merlin FERM domain with PIP2 and PIP5Kα, respectively, PIP5Kα-mediated local PIP2 synthesis at the PM may thus induce synergistic activation of Merlin-dependent LATS1 signaling. We found that the interactions of PIP5Kα with Merlin and LATS1 were much stronger in dense and starved cells. These cell density- and nutrient-mediated alterations suggest that assembly and disassembly of PIP5Kα, Merlin, and LATS1 interactions constitute important factors for Hippo pathway regulation.

The FERM domain is present in various actin cytoskeletal proteins, including ezrin, radixin, and moesin, mediating their attachment to the PM via PIP2 binding and is also involved in protein interactions with its binding partners [16, 30, 60]. Similar to Merlin, the cytoskeletal protein talin binds to PIP2 and PIP5Kγ through its FERM domain at focal adhesions [61, 62]. PIP2-binding proteins also bind to PIP5K because of their close proximity to the sites where PIP2 is generated by PIP5K [32]. For example, the clathrin adaptor protein AP-2 binds to PIP2 and PIP5K at endocytic membranes [37, 63]. Here, we showed that the F1 subdomain of the Merlin FERM domain is mainly responsible for binding to PIP5Kα. Alternatively, the F2 subdomain mediates Merlin binding to LATS1 [18]. In addition, several Merlin mutants have been developed that abolish its PIP2 binding property, incorporating six (K79N, K80N, K269N, E270N, K278N, and K279N), four (T59V, W60E, R309Q, and R310Q), or a combination of all 10 point mutations, identifying that the key residues for PIP2 binding resided in the F1 and F3 subdomains [19, 20, 34]. Together with these findings, our results support that the Merlin FERM domain serves as a binding scaffold that accommodates PIP5Kα, PIP2, and LATS1 in a cooperative manner.

We further found that PIP5Kα deficiency resulted in aberrantly elevated YAP/TAZ signaling at high cell density and in serum-stimulated cells after starvation, as shown by the relatively low levels of YAP phosphorylation and high levels of TAZ protein and YAP/TAZ target gene induction. Moreover, increased protein stability and decreased degradative ubiquitination of PIP5Kα at high cell densities led to enhanced PIP5Kα protein levels compared to those at low cell densities. Thus, it is likely that the increase in PIP2 levels at high cell densities results from enhanced PIP5Kα levels. Multiple E3 ubiquitin ligases and deubiquitinases are involved in controlling the Hippo-YAP/TAZ pathway through ubiquitin-mediated degradation of various Hippo signaling proteins [64–66]. At present, however, it remains unclear which E3 ubiquitin ligase and/or deubiquitinase regulates PIP5Kα ubiquitination and protein stability. Upon serum stimulation, the time course of changes in PIP2 levels correlated with those in YAP phosphorylation levels. Several growth factor receptor signaling pathways involve the activation of PIP2-specific PLCs, such as PLCγ1, which catalyzes PIP2 hydrolysis [67]. Thus, it is plausible that the initial decline in PIP2 levels upon serum stimulation is attributable to PLC activation, with PIP5Kα mediating the subsequent recovery of PIP2 levels. Overall, our results suggest that changes in PIP2 levels can function as switch signals for cell density- and nutrient-dependent Hippo pathway regulation.

YAP/TAZ signaling is involved in many critical cellular processes, including tumorigenesis, regeneration, mechano-transduction, angiogenesis, metabolic pathways, and stem cell differentiation [1–3, 9, 68]. Therefore, YAP/TAZ activity is tightly regulated under physiological conditions. Our results indicated that PIP5Kα inhibits YAP/TAZ in the Hippo pathway. In accordance with this, PIP5Kα impeded YAP/TAZ-dependent cell proliferation, likely through Merlin and LATS1 activation. In addition, PIP5Kα promoted the adipogenic differentiation of MSCs, confirming PIP5Kα-driven YAP/TAZ inactivation. In turn, Merlin is a critical tumor suppressor, and its inactivation by loss-of-function mutations in the *NF2* gene causes NF2 syndromes such as Schwannoma, meningioma, and mesothelioma [48, 69]. Our results showed that one such mutant of Merlin (L64P) neither interacted with PIP5Kα and LATS1 nor localized to the PM. Thus, we consider that the association of Merlin with PIP5Kα and PIP2 in the PM environment represents a novel aspect for understanding the pathological mechanisms of NF2. In this regard, it is worth investigating whether other missense mutations in Merlin, especially those in the FERM or F1 subdomain, exhibit similarities with the L64P mutant in terms of PM localization and interaction with PIP5Kα and/or PIP2.

The type I PIP5K family members, PIP5Kα, PIP5Kβ, and PIP5Kγ, mediate their own unique functions but can also show some overlapping functions [27]. Our results suggest that the three PIP5K isoforms may regulate the Hippo-YAP/TAZ pathway in a similar mechanism. This notion can also be supported by our findings that PIP5Kα interacted with Merlin through the relatively well-conserved catalytic domain among the PIP5K family members although a specific binding motif within the catalytic domain is not yet identified. Previously, Hong et al. reported that overexpression of PIP5Kα or PIP5Kγ, but not of PIP5Kβ, increased LATS1 and YAP phosphorylations [34]. Our results are similar to, but somewhat different from, the previous results in that PIP5Kβ also increased LATS1 phosphorylation and inhibited YAP activity. The individual PIP5K isoforms may play a similar or distinct role in the regulation of Hippo-YAP/TAZ pathway depending on cell types, their expression levels, and/or different Hippo signaling conditions.

On the other hand, it has been shown that PIP5Kα can act upstream of phosphatidylinositol 3-kinase (PI3K)/Akt signaling pathway that promotes cell survival and proliferation [35, 70, 71]. PIP2 is used as a substrate for PI3K-mediated production of phosphatidylinositol 3,4,5-trisphosphate (PIP3), the important lipid to stimulate Akt [72]. Thus, PIP5Kα is recognized as a novel antitumor target especially in the case of breast and prostate cancer [70, 71]. Considering these, PIP5Kα-dependent PIP2 production can be implicated in both Hippo pathway and PI3K/Akt pathway. As the two signaling pathways oppositely regulate oncogenic cell proliferation, we assume that PIP5Kα may bidirectionally modulate cell growth and proliferation depending on specified cellular contexts and certain changes in PM microenvironment, thereby playing as a homeostatic regulator. In fact, it is also known that PIP2 promotes activation of phosphatase and tensin homolog (PTEN) that is an established tumor suppressor to antagonize PI3K/Akt signaling through hydrolyzing PIP3 [73, 74]. In addition, it is likely that various upstream factors regulating PIP5Kα catalytic activity and/or expression levels influence PIP5Kα-mediated control of cell proliferation.

In conclusion, we demonstrated that PIP5Kα contributes to activation of the Hippo pathway through PIP2-dependent Merlin and LATS1 activation, leading to YAP/TAZ inhibition and thereby restricting cell proliferation and facilitating adipogenic differentiation. Moreover, PIP5Kα has a regulatory function under cell density and serum stimulation conditions associated with Hippo-YAP/TAZ pathway activity, and plays a role, together with PIP2, in coordinating the interaction of Merlin with LATS1 at the PM. Thus, our results suggest that PIP5Kα-dependent PIP2 production serves as a point of regulation for the canonical Hippo-YAP/TAZ pathway through mediating activation of Merlin and LATS1 at the PM.

## Supplementary Information

**Additional file 1**: **Fig. S1.** Effects of PIP5Kα KO or overexpression on the Hippo-YAP/TAZ pathway. **Fig. S2.** PIP5Kα enhances the degradative ubiquitination of TAZ. **Fig. S3.** Reduction in PIP2 levels in PIP5Kα-deficient and PIP5Kα KD-transfected cells. **Fig. S4.** PIP5Kα acts as an inhibitor of YAP/TAZ in a PIP2-dependent manner. **Fig. S5.** Effects of PIP5Kα KO or KD mutant on PIP5Kβ and PIP5Kγ expression levels. **Fig. S6.** Effects of the type I PIP5Ks overexpression on Hippo-YAP/TAZ pathway. **Fig. S7.** Effects of PIP5Kα KO on phosphorylations of MAPK family members and Akt. **Fig. S8.** Aberrant YAP/TAZ activation in PIP5Kα KO cells under different cell density and serum stimulation conditions. **Fig. S9.** Dislocation of LATS1 from the PM upon serum stimulation. **Fig. S10.** Lack of binding affinity of PIP5Kα N-terminal and CT domains for Merlin and LATS1. **Fig. S11.** PIP5Kα truncated forms colocalize with Merlin and LATS1, and PIP5Kα colocalization with Merlin FERM domain. **Fig. S12.** Loss of interaction and colocalization of the Merlin L64P mutant with PIP5Kα. **Fig. S13.** Effects of Merlin and LATS1 ablation on PIP5Kα-induced phosphorylation of LATS1 and YAP. **Fig. S14.** Proposed model for a potential role of PIP5Kα in regulation of the Hippo pathway. **Table S1.** qRT-PCR primers used in this study.

## Data Availability

All data generated or analyzed during this study are included in this published article and its supplementary information file.

## Abbreviations

ANKRD1: Ankyrin repeat domain-containing protein 1
CHX: Cycloheximide
CT: C-terminal
CTGF: Connective tissue growth factor
CYR61: Cysteine-rich angiogenic inducer 61
FERM: Band 4.1/Ezrin/Radixin/Moesin
GFP: Green fluorescent protein
GST: Glutathione *S*-transferase
IP: Immunoprecipitation
KD: Kinase-dead
KO: Knockout
LATS1: Large tumor suppressor kinase 1
mRFP: Monomeric red fluorescent protein
NF2: Neurofibromatosis type 2
PH: Pleckstrin homology
PIP2: Phosphatidylinositol 4,5-bisphosphate
PIP5Kα: Phosphatidylinositol 4-phosphate 5-kinase α
PLC: Phospholipase C
PM: Plasma membrane
qRT-PCR: Quantitative real-time reverse transcription PCR
TAZ: Transcriptional co-activator with PDZ-binding motif
WB: Western blotting
WT: Wild-type
YAP: Yes-associated protein
YFP: Yellow fluorescent protein
