# Supplementary material for: Lipid kinase PIP5Kα contributes to Hippo pathway activation via interaction with Merlin and by mediating plasma membrane targeting of LATS1
Source: Cell Commun Signal. 2023 Jun 19;21:149. doi: 10.1186/s12964-023-01161-w (PMC10278285; doi:10.1186/s12964-023-01161-w)
Supplement: Supplementary file 2 — Additional file 1: Fig. S1. Effects of PIP5Kα KO or overexpression on the Hippo-YAP/TAZ pathway. Fig. S2. PIP5Kα enhances the degradative ubiquitination of TAZ. Fig. S3. Reduction in PIP2 levels in PIP5Kα-deficient and PIP5Kα KD-transfected cells. Fig. S4. PIP5Kα acts as an inhibitor of YAP/TAZ in a PIP2-dependent manner. Fig. S5. Effects of PIP5Kα KO or KD mutant on PIP5Kβ and PIP5Kγ expression levels. Fig. S6. Effects of the type I PIP5Ks overexpression on Hippo-YAP/TAZ pathway. Fig. S7. Effects of PIP5Kα KO on phosphorylations of MAPK family members and Akt. Fig. S8. Aberrant YAP/TAZ activation in PIP5Kα KO cells under different cell density and serum stimulation conditions. Fig. S9. Dislocation of LATS1 from the PM upon serum stimulation. Fig. S10. Lack of binding affinity of PIP5Kα N-terminal and CT domains for Merlin and LATS1. Fig. S11. PIP5Kα truncated forms colocalize with Merlin and LATS1, and PIP5Kα colocalization with Merlin FERM domain. Fig. S12. Loss of interaction and colocalization of the Merlin L64P mutant with PIP5Kα. Fig. S13. Effects of Merlin and LATS1 ablation on PIP5Kα-induced phosphorylation of LATS1 and YAP. Fig. S14. Proposed model for a potential role of PIP5Kα in regulation of the Hippo pathway. Table S1. qRT-PCR primers used in this study. [file 12964_2023_1161_MOESM1_ESM.pdf]

Supplementary Information

Additional file 1

- **Supplementary Figures S1–S14**
- **Supplementary Table S1**

Lipid kinase PIP5Kα contributes to Hippo pathway activation via interaction with Merlin and by mediating plasma membrane targeting of LATS1

Truc Phan Hoang Le[1], Nga Thi Thanh Nguyen[1], Duong Duy Thai Le[1], Muhammad Ayaz Anwar[2] and Sang Yoon Lee[1,3,*]

[1] Department of Biomedical Sciences, Ajou University Graduate School of Medicine, Suwon, Gyeonggi 16499, Republic of Korea

[2] Department of Applied Chemistry, Kyung Hee University International Campus, Yongin, Gyeonggi 17104, Republic of Korea

[3] Institute of Medical Science, Ajou University School of Medicine, Suwon, Gyeonggi 16499, Republic of Korea

* Correspondence:
Sang Yoon Lee
sangyoon@ajou.ac.kr

Supplementary Figure 1

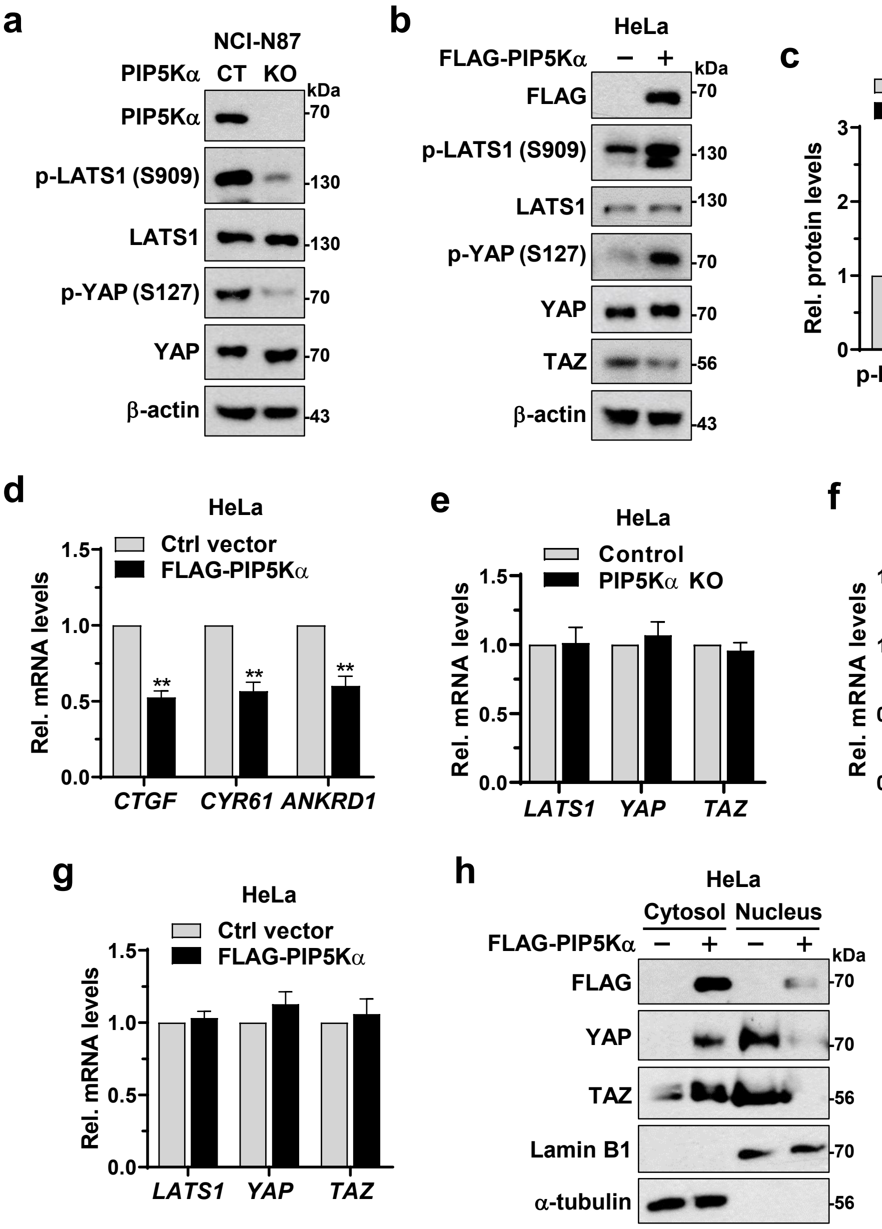

Fig. S1 Effects of PIP5Kα KO or overexpression on the Hippo-YAP/TAZ pathway. Control and PIP5Kα KO NCI-N87 cell lysates (**a**) and HeLa cell lysates following control vector or FLAG-PIP5Kα transfection (**b**) were immunoblotted using the indicated antibodies. **c** Relative quantification of LATS1 and YAP phosphorylation and TAZ protein levels in (**b**) ($n = 4$). **d** YAP/TAZ target gene induction as measured by qRT-PCR analysis under the same condition as in (**b**) ($n = 4$). *LATS1*, *YAP*, and *TAZ* mRNA levels in control and PIP5Kα KO HeLa (**e**) and HEK293 (**f**) cells, and FLAG-PIP5Kα-transfected HeLa cells (**g**), as analyzed by qRT-PCR ($n = 3$). (**h**) Cytosolic and nuclear fractions prepared under the same condition as in (**b**) were analyzed by WB using the indicated antibodies. Values in the graphs represent the means ± SEM. $^{*}p < 0.05$, $^{**}p < 0.01$

Supplementary Figure 2

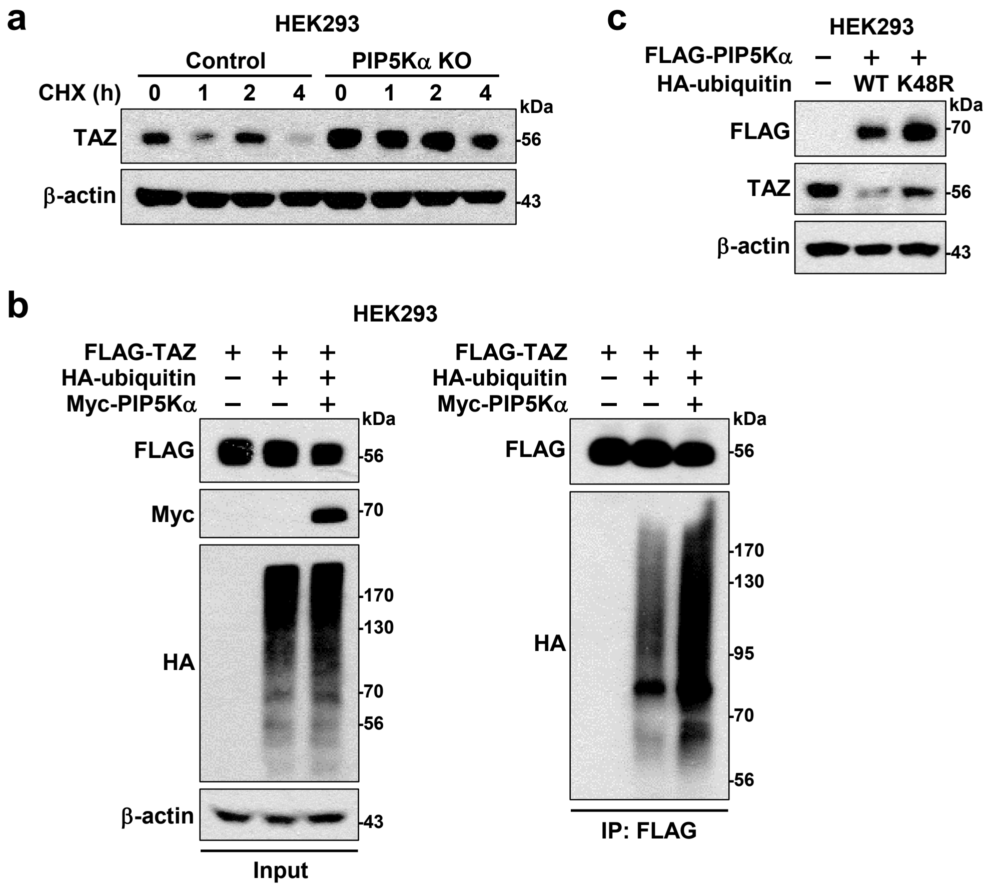

Fig. S2 PIP5Kα enhances the degradative ubiquitination of TAZ. **a** Control and PIP5Kα KO HEK293 cells were treated with CHX (100 μM) for the indicated times and cell lysates were immunoblotted using an anti-TAZ antibody. **b** FLAG-TAZ was cotransfected into HEK293 cells with HA-ubiquitin and/or Myc-PIP5Kα, as indicated. FLAG IP products and cell lysates were analyzed by WB using the indicated antibodies. **c** WB analysis of HEK293 cell lysates following cotransfection with FLAG-PIP5Kα and HA-ubiquitin (WT or K48R)

Supplementary Figure 3

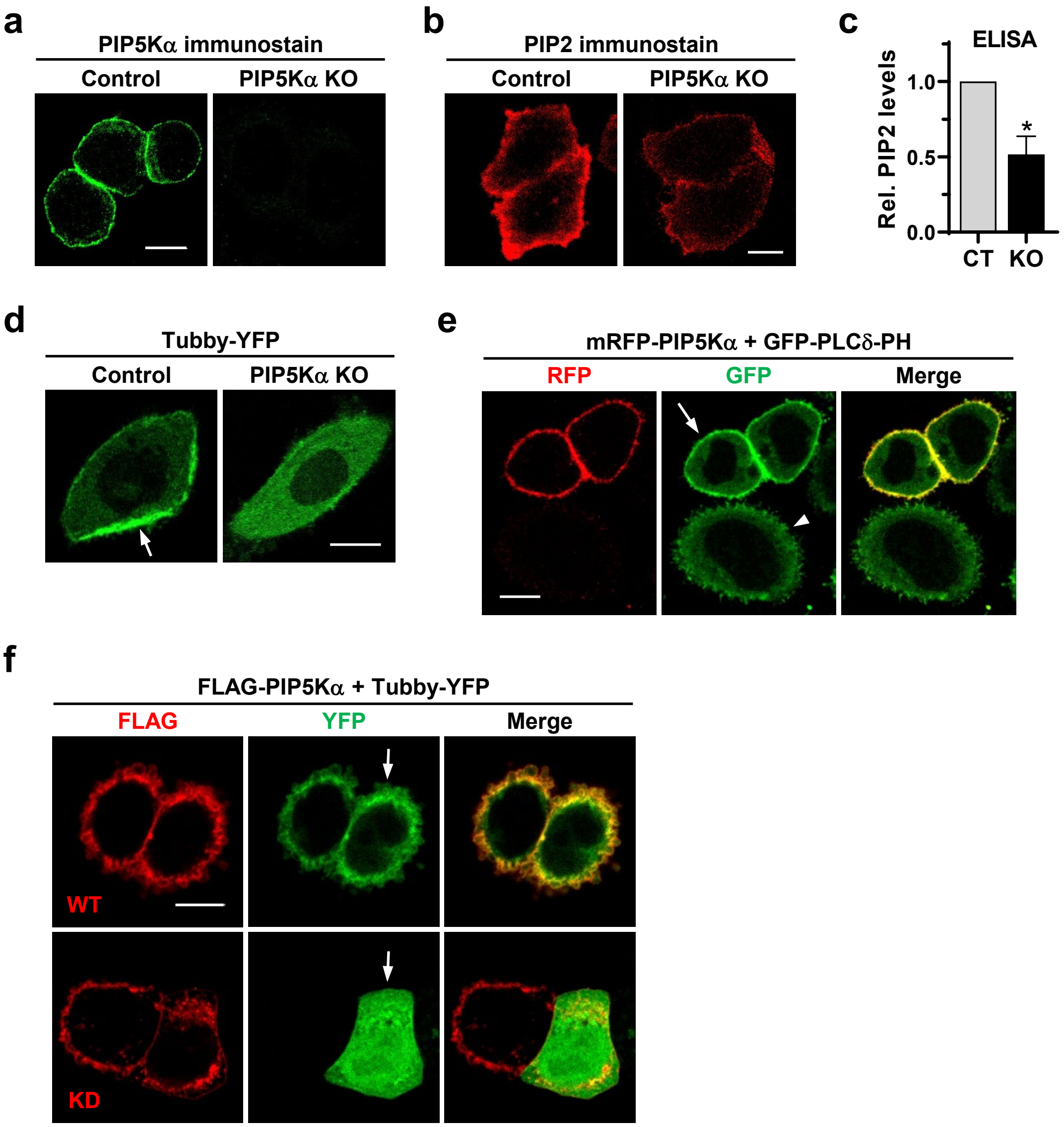

Fig. S3 Reduction in PIP2 levels in PIP5Kα-deficient and PIP5Kα KD-transfected cells. Representative confocal images of immunostained PIP5Kα (**a**) and PIP2 (**b**) in control and PIP5Kα KO HeLa cells. **c** Relative quantification of PIP2 levels in control and PIP5Kα KO HeLa cells as measured using a PIP2 ELISA kit ($n = 3$). Values in the graph represent the means ± SEM. *$p < 0.05$. **d** Representative confocal images of Tubby-YFP transfected into control and PIP5Kα KO HeLa cells. The arrow indicates a relatively high level of PIP2. **e** Representative confocal images of HeLa cells cotransfected with mRFP-PIP5Kα and GFP-PLCδ-PH. The arrow and arrowhead indicated high and low levels of PIP2 in the mRFP-positive and -negative cells, respectively. **f** Representative confocal images of HeLa cells cotransfected with Tubby-YFP and FLAG-PIP5Kα WT or KD. Cells were immunostained with an anti-FLAG antibody, followed by Alexa Fluor 594-labeled secondary antibody. The arrows indicate high and low levels of PIP2 in the WT- and KD-expressing cells. Scale bars, 10 μm

Supplementary Figure 4

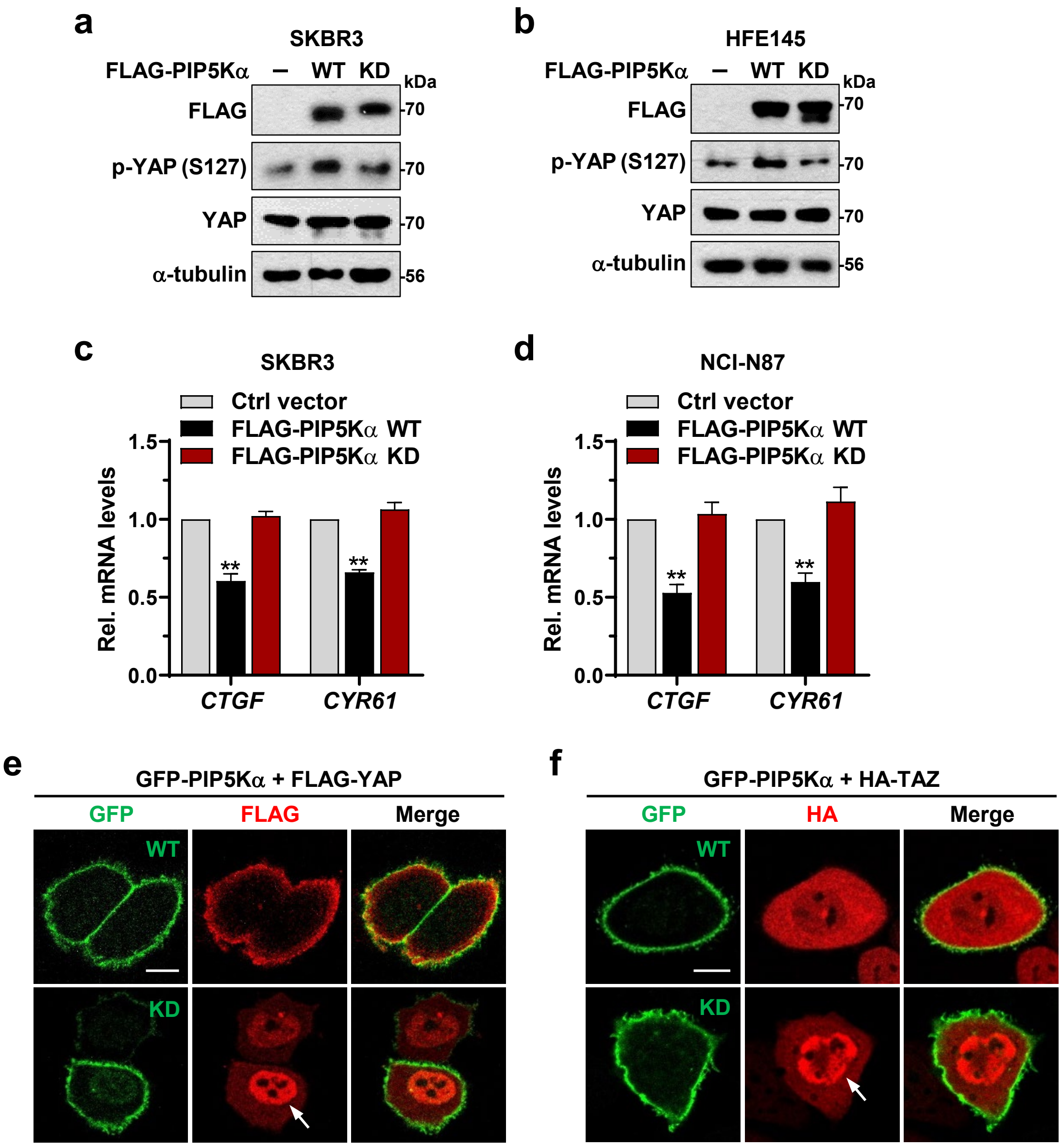

Fig. S4 PIP5Kα acts as an inhibitor of YAP/TAZ in a PIP2-dependent manner. SKBR3 (**a**) and HFE145 (**b**) cells were transfected with control vector, or FLAG-PIP5Kα WT or KD, and the resulting cell lysates were analyzed by WB using the indicated antibodies. *CTGF* and *CYR61* mRNA levels in SKBR3 (**c**) and NCI-N87 (**d**) cells transfected with FLAG-PIP5Kα WT or KD were analyzed using qRT-PCR and quantified relative to the levels upon control vector transfection ($n = 3$). Values in the graphs represent the means ± SEM. $^{**}p < 0.01$. **e**, **f** GFP-PIP5Kα WT or KD was cotransfected into HeLa cells with FLAG-YAP or HA-TAZ, as indicated. FLAG and HA were immunostained *via* their primary antibodies and Alexa Fluor 594-labeled secondary antibody. The arrows indicate nuclear localization of FLAG-YAP and HA-TAZ. Scale bars, 10 μm

Supplementary Figure 5

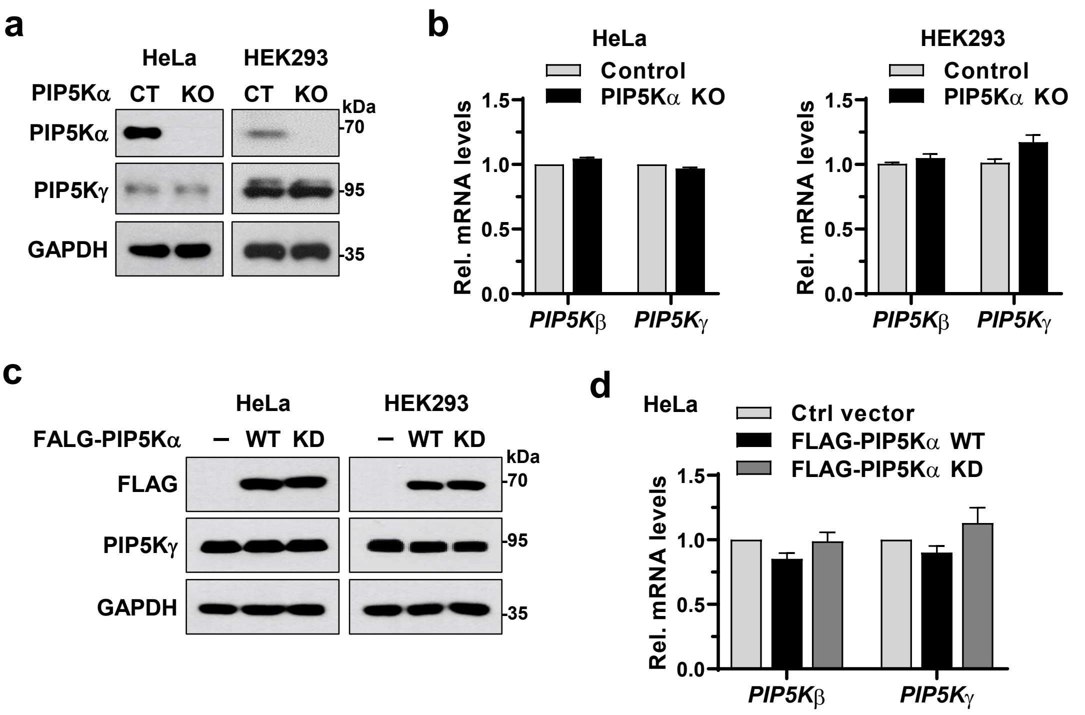

Fig. S5 Effects of PIP5Kα KO or KD mutant on PIP5Kβ and PIP5Kγ expression levels. **a** Control and PIP5Kα KO HeLa and HEK293 cell lysates were immunoblotted using anti-PIP5Kα and anti-PIP5Kγ antibodies. **b** PIP5Kβ and PIP5Kγ mRNA levels in PIP5Kα KO cells in (**a**) were analyzed by qRT-PCR and quantified relative to those in control cells, respectively (*n* = 3). HeLa (**c**, **d**) or HEK293 (**c**) cells were transfected with control vector, FLAG-PIP5Kα WT, or its KD form. **c** Resulting cell lysates were analyzed by WB using the indicated antibodies. **d** Relative quantification of PIP5Kβ and PIP5Kγ mRNA levels analyzed by qRT-PCR (*n* = 6)

Supplementary Figure 6

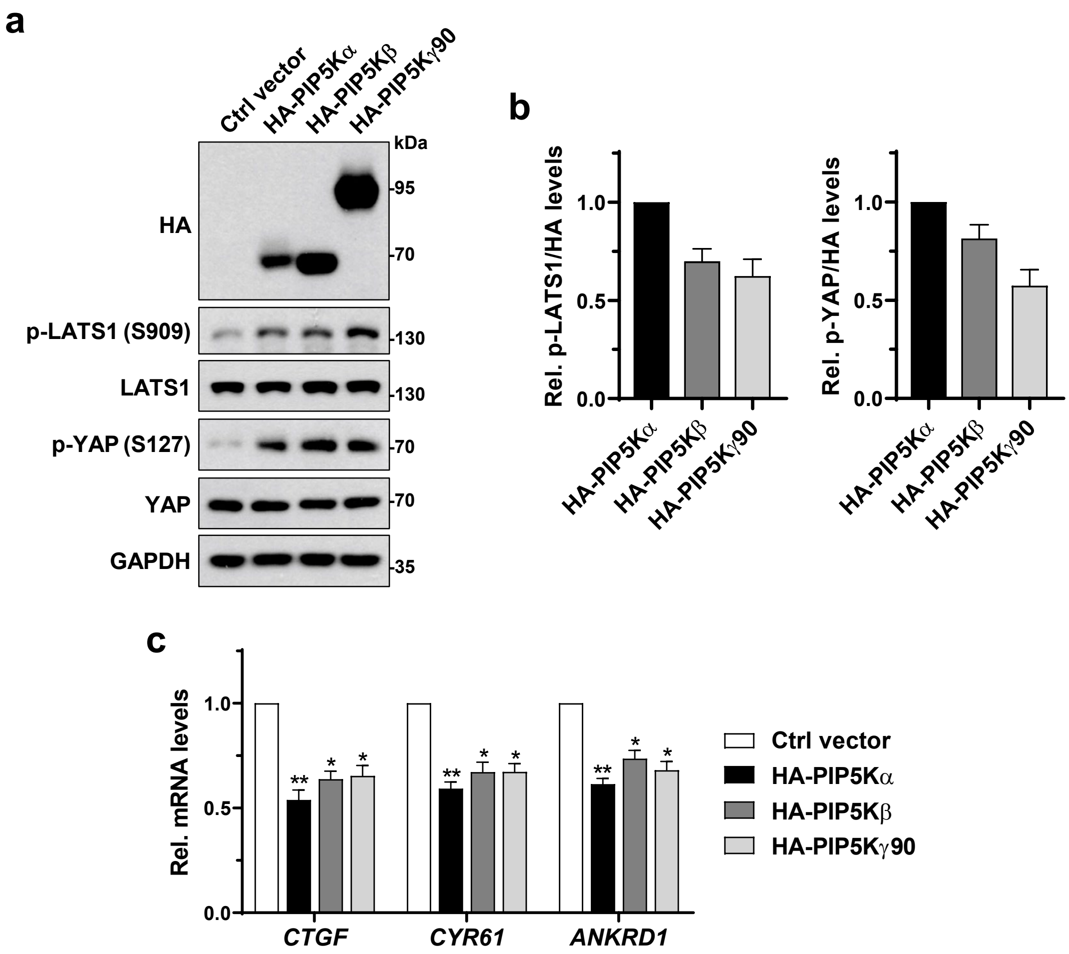

Fig. S6 Effects of the type I PIP5Ks overexpression on Hippo-YAP/TAZ pathway. **a**, **c** HEK293 cells were transfected with control vector, HA-tagged PIP5Kα, PIP5Kβ, or PIP5Kγ90 expression plasmids. **a** Resulting cell lysates were analyzed by WB using the indicated antibodies. **b** Phosphorylation levels of LATS1 and YAP in (**a**) were normalized to respective HA-PIP5K expression levels, and quantified relative to those in HA-PIP5Kα transfection ($n = 3$). **c** *CTGF*, *CYR61*, and *ANKRD1* mRNA levels were analyzed by qRT-PCR and quantified relative to those in control vector transfection ($n = 3$). Values in the graphs represent the means ± SEM. $^{*}p < 0.05$, $^{**}p < 0.01$

Supplementary Figure 7

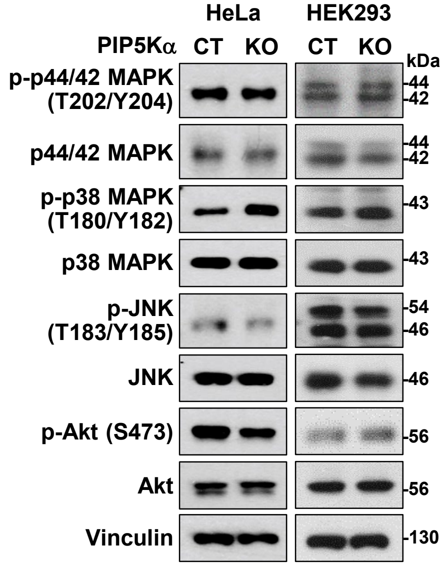

Fig. S7 Effects of PIP5Kα KO on phosphorylations of MAPK family members and Akt. Control and PIP5Kα KO HeLa and HEK293 cell lysates were analyzed by WB using the indicated antibodies for detecting total and phosphorylation levels of p44/42 MAPK, p38 MAPK, JNK, or Akt

Supplementary Figure 8

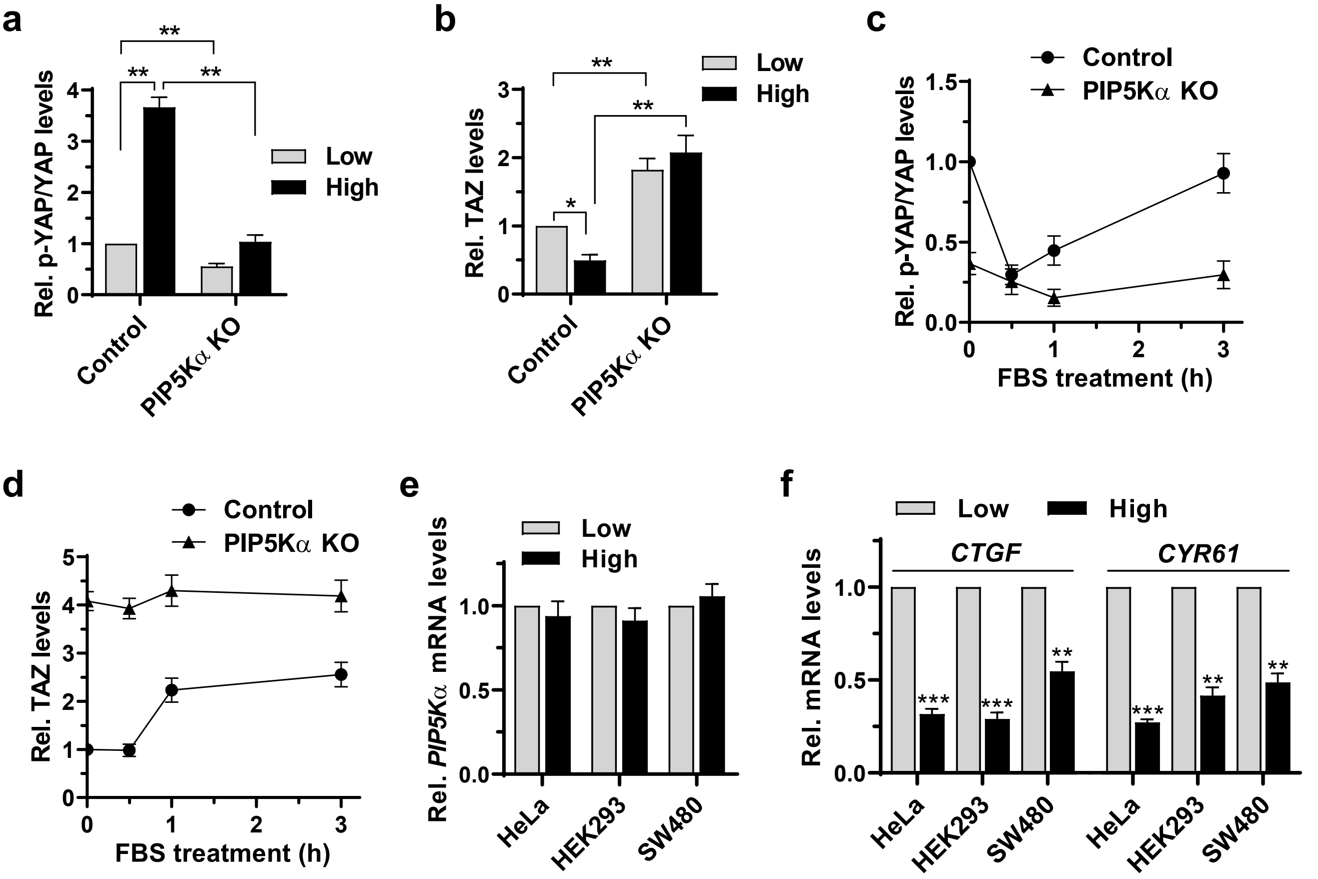

Fig. S8 Aberrant YAP/TAZ activation in PIP5Kα KO cells under different cell density and serum stimulation conditions. **a**, **b** Quantification of phosphorylation/total YAP ratios and TAZ protein levels in Fig. 2a relative to those in control cells at low density ($n = 3$). **c**, **d** Quantification of phosphorylation/total YAP ratios and TAZ protein levels in Fig. 2c relative to those in control cells at the zero-time point ($n = 3$). *PIP5Kα* (**e**) and *CTGF* and *CYR61* (**f**) mRNA levels in HeLa, HEK293, and SW480 cells at low and high density were analyzed using qRT-PCR and quantified relative to those at low density ($n = 4$). Values in the graphs represent the means ± SEM. $^{*}p < 0.05$, $^{**}p < 0.01$, $^{***}p < 0.001$

Supplementary Figure 9

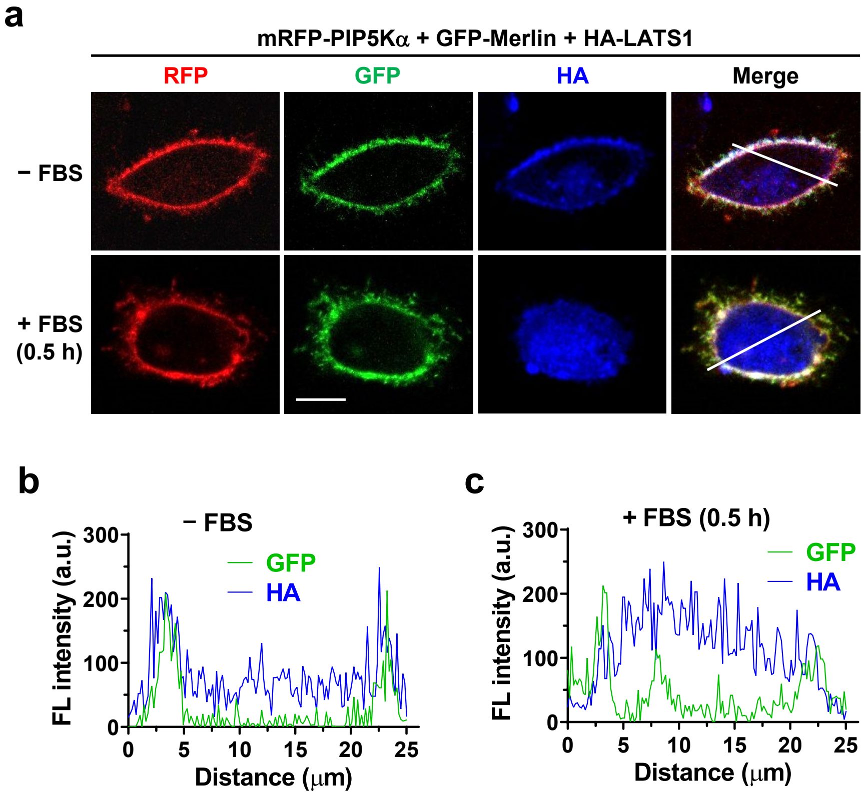

Fig. S9 Dislocation of LATS1 from the PM upon serum stimulation. **a** HeLa cells cotransfected with mRFP-PIP5Kα, GFP-Merlin, and HA-LATS1 were treated with or without FBS for 30 min. HA-LATS1 was visualized by HA immunostaining, followed by staining with an Alexa Fluor 350-labeled secondary antibody. Representative images of mRFP, GFP, and HA immunofluorescence were obtained using confocal microscopy. Scale bar, 10 μm. **b**, **c** Profile graphs of GFP and HA fluorescent (FL) intensities along the lines in (**a**), as acquired using Zeiss ZEN imaging software

Supplementary Figure 10

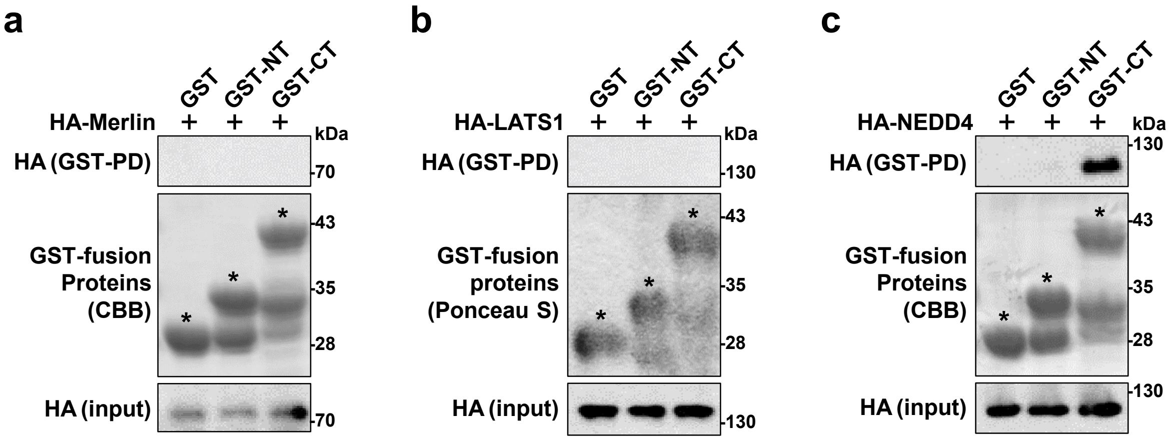

Fig. S10 Lack of binding affinity of PIP5Kα N-terminal and CT domains for Merlin and LATS1. GST alone and GST-fusion proteins with the N-terminal (NT) or CT domain of PIP5Kα were mixed with cell lysates prepared from HEK293 cells transfected with HA-Merlin (**a**), HA-LATS1 (**b**), or HA-NEDD4 (**c**). Cell lysates (input) and GST pull-down (PD) samples were immunoblotted using an anti-HA antibody. The asterisk indicates GST and the GST-fusion proteins detected using Coomassie brilliant blue (CBB) or Ponceau S staining

Supplementary Figure 11

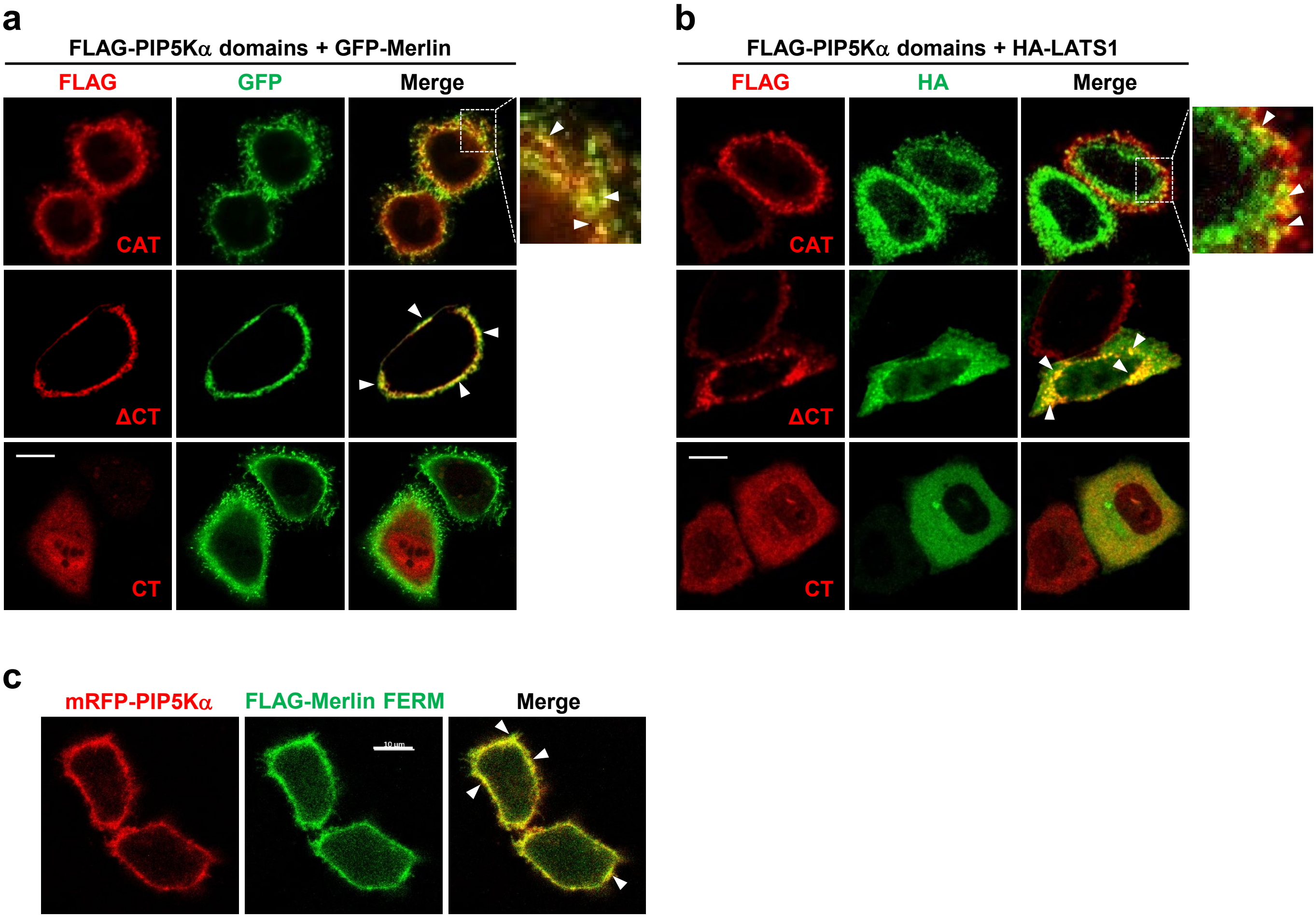

Fig. S11 PIP5Kα truncated forms colocalize with Merlin and LATS1, and PIP5Kα colocalization with Merlin FERM domain. Representative confocal images of the FLAG-tagged catalytic (CAT) domain, ΔCT form, or CT domain of PIP5Kα cotransfected into HeLa cells together with GFP-Merlin (**a**) or HA-LATS1 (**b**), as indicated. The FLAG-tagged proteins and HA-LATS1 were visualized using FLAG and HA immunostaining, followed by staining with Alexa Fluor 594- and 488-labeled secondary antibodies, respectively. The arrowheads and those in the magnified area indicate colocalization of GFP-Merlin or HA-LATS1 with FLAG-PIP5Kα CAT and FLAG-PIP5Kα ΔCT. **c** Representative confocal images of HeLa cells cotransfected with mRFP-PIP5Kα and the FLAG-Merlin FERM domain. The arrowheads indicate enrichment of both proteins in the PM. Scale bars, 10 μm

Supplementary Figure 12

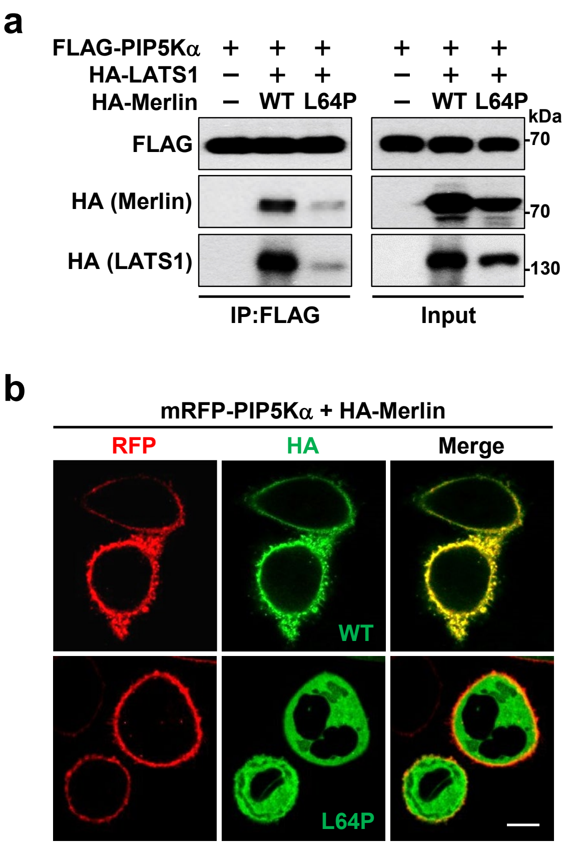

Fig. S12 Loss of interaction and colocalization of the Merlin L64P mutant with PIP5Kα. **a** HEK293 cells were cotransfected with FLAG-PIP5Kα, HA-LATS1, and HA-Merlin WT or L64P, as indicated. Cell lysates and FLAG-IP products were then immunoblotted using anti-FLAG and anti-HA antibodies. **b** Representative confocal images of HeLa cells cotransfected with mRFP-PIP5Kα and HA-Merlin WT or L64P. Cells were immunostained with an anti-HA antibody and Alexa Fluor 488-labeled secondary antibody. Scale bar, 10 μm

Supplementary Figure 13

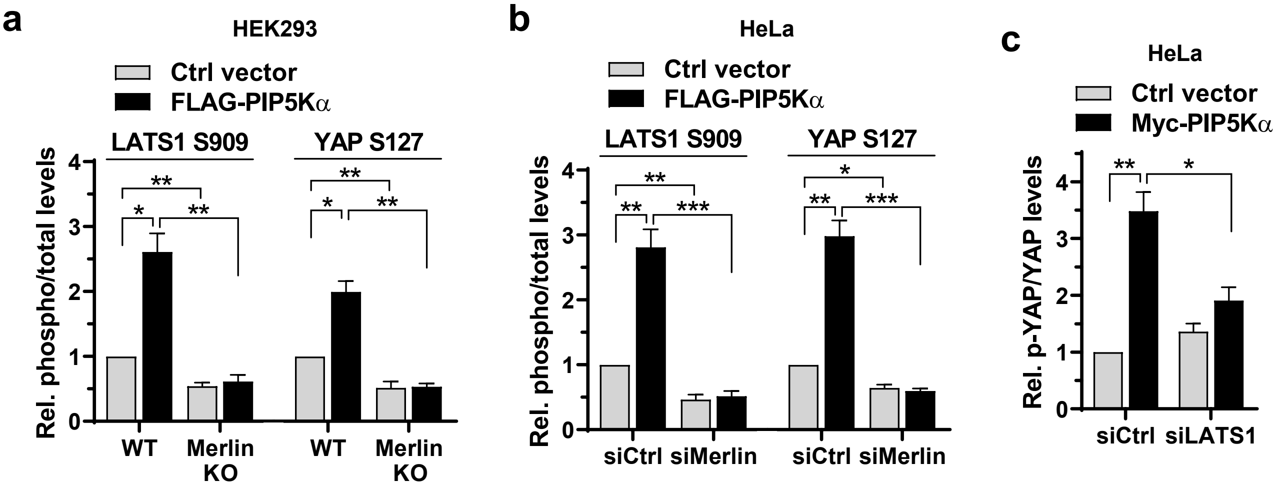

Fig. S13 Effects of Merlin and LATS1 ablation on PIP5Kα-induced phosphorylation of LATS1 and YAP. **a**–**c** Quantification of the phosphorylation/total ratios of LATS1 and YAP in Fig. 6a–c, respectively, relative to those following control vector and/or siRNA transfection (**a**, $n = 5$; **b**, $n = 3$; **c**, $n = 3$). Values in the graphs represent the means ± SEM. *$p < 0.05$, **$p < 0.01$, ***$p < 0.001$

Supplementary Figure 14

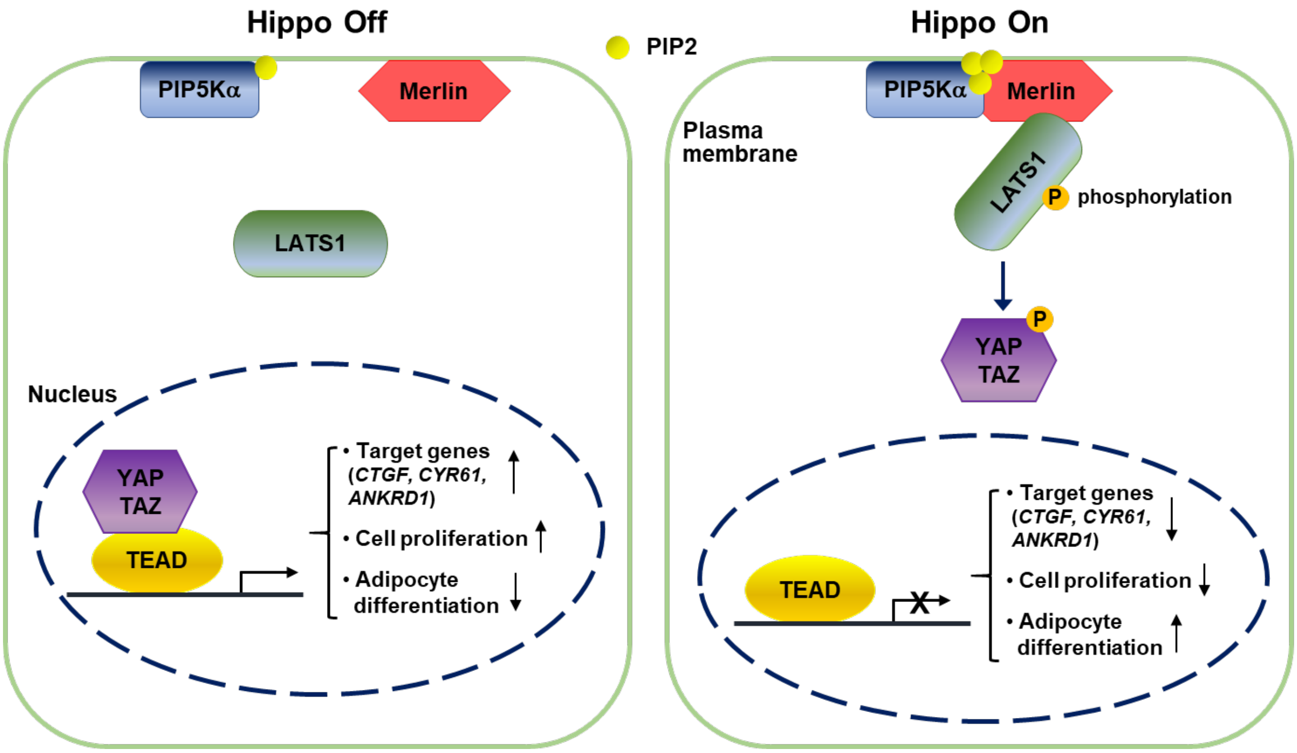

Fig. S14 Proposed model for a potential role of PIP5Kα in regulation of the Hippo pathway. PIP5Kα contributes to activation of the Hippo pathway through interacting with Merlin in the PM and recruiting LATS1 to the PIP2-rich PM, thereby regulating YAP/TAZ-dependent cell proliferation and differentiation.

Supplementary Table

Table S1 qRT-PCR primers used in this study

Gene	Sequences
human CTGF	Forward, 5'-CCTGCAGGCTAGAGAAGCAG-3'<br>Reverse, 5'-TGGAGATTTTGGGAGTACGG-3'
human CYR61	Forward, 5'-AAGAAACCCGGATTTGTGAG-3'<br>Reverse, 5'-GCTGCATTTCTTGCCCTTT-3'
human ANKRD1	Forward, 5'-TTTGGCAATTGTGGAGAAGTTA-3'<br>Reverse, 5'-AAACATCCAGGTTTCCTCCA-3'
human PIP5Kα	Forward, 5'-GGCACAAGTGACAACAAAGG-3'<br>Reverse, 5'-CAAAGGTGGAGTCTGAGGTAAA-3'
human PIP5Kβ	Forward, 5'-AGAAGCTCCCTGGCTAC-3'<br>Reverse, 5'-GGCATGTCCTGCATGAAGTC-3'
human PIP5Kγ	Forward, 5'-CCGTCTAAGAAACGGTGCAA-3'<br>Reverse, 5'-TGATGGAGTGCTAGGGACCA-3'
human GAPDH	Forward, 5'-AGGGCTGCTTTTAACTCTGGT-3'<br>Reverse, 5'-CCCCACTTGATTTTGGAGGGA-3'
mouse Pparg	Forward, 5'-AGGAAAGACAACGGACAAATCACC-3'<br>Reverse, 5'-ATTCGGATGGCCACCTCTTTGC-3'
mouse Fabp4	Forward, 5'-GATGAAATCACCGCAGACGACA-3'<br>Reverse, 5'-ATTGTGGTCGACTTTCCATCCC-3'
mouse Ucp1	Forward, 5'-GCATTCAGAGGCAAATCAGC-3'<br>Reverse, 5'-GCCACACCTCCAGTCATTAAG-3'
mouse GAPDH	Forward, 5'-ACCCAGAAGACTGTGGATGG-3'<br>Reverse, 5'-CACATTGGGGGTAGGAACAC-3'
